# The effects of physical activity interventions on stress-related psychological and physiological outcomes in children and adolescents: a systematic review and meta-analysis

**DOI:** 10.3389/fpsyg.2026.2000159

**Published:** 2026-10-21

**Authors:** Liang Chen, Jia Zhang, Bo Wang, Zhongling Gao

**Affiliations:** School of Physical Education and Health, Linyi University, Linyi, China

**Keywords:** children and adolescents, exercise, perceived stress, physical activity, physiological outcomes, psychological health

## Abstract

**Objective:**

This systematic review and meta-analysis aimed to evaluate the effects of physical activity interventions on stress-related psychological and physiological outcomes in children and adolescents, including direct or subjective stress-related measures, related psychological health outcomes, and physiological indicators potentially associated with stress regulation.

**Methods:**

PubMed, Web of Science, Embase, and the Cochrane Central Register of Controlled Trials (CENTRAL) were systematically searched from database inception to September 8, 2026. The Cochrane Risk of Bias 2 (RoB 2) tool was used to assess the risk of bias in randomized controlled trials, while the Risk Of Bias In Non-randomized Studies—of Interventions (ROBINS-I) tool was used for non-randomized intervention studies; the certainty of evidence for the primary outcomes was assessed using the Grading of Recommendations Assessment, Development and Evaluation (GRADE) framework. Meta-analyses were conducted using a random-effects model, with pooled effects expressed as standardized mean differences (Hedges’ g) or mean differences (MDs), as appropriate for each outcome.

**Results:**

A total of 35 controlled studies were included, comprising 32 randomized controlled trials and 3 non-randomized intervention studies. Primary pooled estimates favored lower perceived/general stress (Hedges’ *g* = −0.28, 95% CI: −0.48 to −0.08, *p* = 0.006, *I*^2^ = 49.9%, τ^2^ = 0.030) and depressive symptoms (Hedges’ *g* = −0.53, 95% CI: −1.04 to −0.02, *p* = 0.041, *I*^2^ = 94.8%, τ^2^ = 0.809), with statistically significant pooled differences also observed for resting systolic blood pressure, resting diastolic blood pressure, and HRV-LF/HF. No statistically significant effects were observed for the remaining psychological or physiological outcomes. Certainty was low for systolic blood pressure and very low for the other statistically significant outcomes. Depressive symptom, diastolic blood pressure, and LF/HF findings were sensitive to the inferential method.

**Conclusion:**

Low-certainty evidence suggested a possible small reduction in resting systolic blood pressure; possible reductions in other outcomes were supported by very-low-certainty evidence. Overall, certainty was very low for most outcomes, and the findings should be regarded as tentative. Several findings were sensitive to analytical choices or individual studies. The non-specificity of related symptoms and resting physiological measures limits inferences about stress regulation. Further high-quality RCTs are warranted.

**Systematic review registration:**

The study was prospectively registered in the PROSPERO database on September 7, 2026 (registration number: CRD420261499943).

## 1 Introduction

Childhood and adolescence represent a critical period for individual physical and psychological development and the formation of social adaptive capacities (Lupien et al., 2009; Sisk and Gee, 2022). During this period, children and adolescents are exposed to multiple sources of stress, including academic demands, interpersonal relationships, family dynamics, and changes in the broader social environment, while their neuroendocrine systems, emotional regulation capacities, and cognitive functions are still undergoing continuous development. Consequently, compared with adults, children and adolescents may exhibit greater sensitivity or variability in their responses to external stressors (Roberts and Lopez-Duran, 2019). In children and adolescents, when exposure to stress is too frequent, prolonged, or accompanied by insufficient regulatory capacity, the stress system may remain persistently activated, potentially resulting in adverse effects on psychological health, cognitive functioning, and physical health (Lupien et al., 2009; Oldehinkel and Bouma, 2011). In recent years, increasing attention has been directed toward stress-related problems among children and adolescents, and identifying safe, accessible, and sustainable approaches to supporting stress management and related health outcomes has become an important priority in promoting child and adolescent health.

Stress responses involve psychological and physiological changes elicited by a perceived or actual challenge to homeostasis (Chrousos, 2009). Resting measurements characterize physiological state, whereas measurements during and after a stressor characterize reactivity and recovery (Laborde et al., 2017). Stress-related psychological and physiological outcomes encompass three related domains: subjective stress experience, associated psychological symptoms, and physiological function. Perceived/general stress reflects the subjective experience of stress. Anxiety, depressive symptoms and fatigue are related psychological health outcomes that may accompany stress but have other determinants and represent distinct constructs. Among physiological indicators, cortisol reflects hypothalamic–pituitary–adrenal (HPA) axis activity, resting heart rate and blood pressure describe cardiovascular state, and heart rate variability (HRV) characterizes aspects of cardiac autonomic regulation (Chrousos, 2009; Laborde et al., 2017). These processes may be involved in adaptation to stress, but the measures are not specific biomarkers of psychological stress. Subjective and physiological responses also need not covary (Campbell and Ehlert, 2012). These domains reflect related but distinct aspects of stress-related health.

Physical activity programs, including structured exercise, are low-cost, readily implementable non-pharmacological approaches to stress management in children and adolescents. Previous studies have suggested that regular participation in exercise may enhance individuals’ capacity to regulate responses to stressors by modulating autonomic nervous system function, influencing hypothalamic–pituitary–adrenal (HPA) axis activity, improving emotional regulation, and promoting sleep and psychosocial adaptation (Lubans et al., 2016; Pascoe et al., 2020). The acute physiological stimuli generated during exercise may promote the development of adaptive responses, whereas long-term regular exercise may improve the regulatory efficiency of stress systems through repeated cycles of physiological stimulation and recovery (Klaperski et al., 2014; Mücke et al., 2018). In addition, the positive emotional experiences, enhanced self-efficacy, social interactions, and accumulation of psychological resources associated with exercise may further alleviate perceived stress and negative emotional states in children and adolescents (Biddle et al., 2019). Exercise may therefore influence subjective stress, related psychological health and physiological function through partly overlapping pathways; improvement in one does not establish improvement in the others.

However, the current evidence regarding the effects of physical activity interventions on stress-related outcomes in children and adolescents remains somewhat inconsistent. On the one hand, substantial differences exist across studies in terms of activity modality, intensity, duration, and intervention period (Mücke et al., 2018). For example, aerobic exercise, resistance exercise, combined exercise, and mind–body exercise may influence stress-regulatory systems through different pathways, and their effects may therefore not be consistent. On the other hand, considerable differences also exist in the outcomes and methods used to assess them, with some studies primarily relying on objective physiological indicators such as cortisol, heart rate variability, heart rate, or blood pressure, whereas others have focused on psychological indicators such as perceived stress, anxiety, or negative affect (Campbell and Ehlert, 2012). This heterogeneity in outcome measures may be an important contributor to the inconsistencies observed in the existing literature. More importantly, physiological and psychological indicators may assess different constructs; an intervention may improve subjective experiences of stress without producing corresponding changes in hypothalamic–pituitary–adrenal (HPA) axis activity or autonomic nervous system function, and vice versa. Therefore, evaluating the effects of physical activity interventions solely from either a psychological or physiological perspective may underestimate or mischaracterize the pattern of effects across stress-related outcomes in children and adolescents.

Previous reviews have to some extent summarized the relationship between physical activity, including exercise, and mental health in children and adolescents; however, joint consideration of subjective stress and related physiological outcomes remains relatively limited (Biddle et al., 2019; Biddle and Asare, 2011). In particular, existing research has often considered stress as one of many mental health outcomes, with relatively little attention given to considering physiological and psychological outcomes together while preserving their conceptual distinctions (Pascoe et al., 2020). Moreover, children and adolescents undergo distinct stages of physiological and psychological development, and differences may exist in the maturation of their stress systems, sources of stress, and capacity for psychological regulation; consequently, the effects of physical activity interventions may also be influenced by factors such as age, activity type, intervention duration, and measurement methods (Carter et al., 2021). Therefore, a comprehensive synthesis of existing randomized controlled trials and relevant intervention studies within a rigorous systematic review framework is warranted to further clarify the effects of physical activity interventions on individual stress-related outcomes and to explore potential factors that may modify these effects.

Accordingly, this study aimed to systematically review and meta-analyze the effects of physical activity interventions on stress-related psychological and physiological outcomes in typically developing children and adolescents. Specifically, this study examined the effects of physical activity interventions on physiological stress-related indicators, including blood cortisol levels, resting heart rate, heart rate variability (HRV; including RMSSD, SDNN, and LF/HF), and resting systolic and diastolic blood pressure, as well as psychological outcomes, including perceived stress, fatigue, anxiety, and depressive symptoms. Outcome-specific estimates may inform stress management and health-promoting physical activity by characterizing changes in subjective stress, related psychological symptoms and physiological function.

## 2 Materials and methods

### 2.1 Registration

This study strictly adhered to the Preferred Reporting Items for Systematic Reviews and Meta-Analyses (PRISMA) guidelines (Page et al., 2021). The study was prospectively registered in the PROSPERO database on September 7, 2026 (registration number: CRD420261499943).

### 2.2 Search strategy

Four electronic databases were systematically searched from database inception to September 8, 2026: PubMed, Web of Science, Embase, and the Cochrane Central Register of Controlled Trials (CENTRAL). The search strategy combined Medical Subject Headings (MeSH) terms and free-text terms and was adapted according to the specific search rules of each database; the complete database-specific strategies are provided in Supplementary methods S1. These four databases were selected to provide broad, complementary coverage of biomedical, behavioral, and exercise-related research and controlled trials. English and Chinese-language reports identified through these searches were eligible for inclusion; dedicated Chinese-language databases were not searched. In addition, the reference lists of the included studies and relevant systematic reviews were manually screened to identify potentially relevant studies that might have been missed by the database searches.

### 2.3 Eligibility criteria and study selection

Physical activity was defined as movement generated by skeletal muscles that expends energy. Exercise was defined as the planned, structured and repetitive subset undertaken to maintain or improve physical fitness. Eligible physical activity interventions encompassed structured exercise and other programs incorporating physical activity. The eligibility criteria were established according to the PICOS framework: (1) Population: Typically developing children and adolescents aged 3–18 years, regardless of sex, with no overweight, obesity, or other diagnosed physical or psychological health conditions; (2) Intervention: Physical activity interventions, including structured exercise (e.g., aerobic exercise, resistance training, high-intensity interval training and combined exercise), sport-based activity, yoga or qigong, and multicomponent school programs; (3) Comparator: Usual activities, no intervention, wait-list control, or other non-exercise interventions; (4) Outcomes: At least one stress-related psychological or physiological outcome, including (a) direct or subjective stress-related outcomes, such as perceived or general stress; (b) related psychological health outcomes, such as anxiety, depressive symptoms, and fatigue; or (c) physiological indicators potentially associated with stress regulation, including cortisol, resting heart rate, resting systolic and diastolic blood pressure, and heart rate variability indices (RMSSD, SDNN, and LF/HF); and (Chrousos, 2009) Study design: Randomized controlled trials or non-randomized intervention studies with a parallel control group.

The exclusion criteria were as follows: (1) participants were outside the age range of 3–18 years, or the study included individuals with overweight, obesity, or other special health conditions without separately reporting data for typically developing children and adolescents; (2) participants had a diagnosed physical or psychological disorder or other health conditions that could potentially affect stress-related outcomes; (3) no eligible physical activity intervention was implemented; (4) an appropriate control group was lacking; (5) no eligible stress-related psychological or physiological outcomes were reported; and (6) observational studies without an appropriate control group, case reports, case series, study protocols, conference abstracts, reviews, editorials, and other non-original publications were excluded.

### 2.4 Study selection

All retrieved records were initially imported into reference management software for deduplication. Subsequently, two researchers independently screened the titles and abstracts of the retrieved records and assessed their potential relevance according to the predefined inclusion and exclusion criteria. Studies that potentially met the eligibility criteria after the initial screening were retrieved in full text and independently assessed for eligibility. For studies whose eligibility could not be determined with certainty, consensus was reached through discussion; if disagreements persisted, a third researcher was consulted to facilitate discussion and make the final decision. The study selection process, including the number and reasons for records excluded at each stage, was documented in accordance with the PRISMA guidelines and presented using a PRISMA flow diagram (Page et al., 2021).

### 2.5 Bias risk and evidence quality assessment

The risk of bias in the included studies was independently assessed by two researchers. For randomized controlled trials, the Cochrane Risk of Bias 2 (RoB 2) tool was used for risk-of-bias assessment (Sterne et al., 2016; Sterne et al., 2019). The certainty of evidence was assessed using the Grading of Recommendations Assessment, Development and Evaluation (GRADE) framework and classified into four levels: high, moderate, low, and very low (Balshem et al., 2011). Any disagreements arising during the assessment process were resolved through discussion, with adjudication by a third researcher when necessary. Imprecision was assessed separately for each outcome by comparing its 95% CI with a *post hoc* distribution-based benchmark of ±0.20 SD, a conventional reference for a small standardized effect rather than a validated outcome-specific minimal important difference. This benchmark addressed uncertainty in standardized magnitude, without assuming equivalent clinical importance across outcomes. An approximate optimal information size of 786 participants was used as supporting information, with identical assumptions for all outcomes (equal independent groups, two-sided *α* = 0.05, 80% power, and a standardized difference of 0.20); the methodological rationale, calculation and outcome-level judgments are provided in Supplementary Table S1.

### 2.6 Data extraction and outcome classification

Data were independently extracted by two researchers using a standardized data-extraction form. Extracted information included study characteristics (first author, publication year, country, and study design), participant characteristics (sample size, age, and sex), intervention and comparator characteristics, outcome measures and assessment instruments, measurement units, and post-intervention data (means, standard deviations, and sample sizes). Supplementary materials were also examined when available. The two researchers independently cross-checked the extracted data against the original publications, and disagreements were resolved through discussion or adjudication by a third researcher.

When discrepancies were identified across the main text, tables, figures, or Supplementary materials, the original report was re-examined. Values were corrected or derived when supported by clearly identifiable information or transparent statistical or unit conversion. When numerical data were reported only in figures, they were extracted when sufficiently precise values could be obtained. For studies reporting multiple intervention arms or multiple eligible measures of the same outcome, data were handled to avoid double-counting participants and statistical dependence. When multiple follow-up time points were reported, the post-intervention assessment at the end of the intervention was prioritized. Post-intervention data were preferred over change-from-baseline data when both were available. Missing standard deviations were derived from other reported statistical information when appropriate; otherwise, the available data were retained only when they could be used with reasonable confidence. Data-processing and conversion procedures were documented and applied consistently across studies.

The PROSPERO registration pre-specified psychological and physiological stress-related outcome domains but did not designate individual outcomes as primary or secondary. The registered outcome scope was retained, with no primary–secondary ranking of individual outcomes. Given the multidimensional nature of stress-related processes, outcomes were classified into three conceptual domains. First, direct or subjective stress-related outcomes included perceived or general stress. Second, related psychological health outcomes included anxiety, depressive symptoms, and fatigue, which may be associated with stress-related processes but represent distinct psychological constructs. Third, physiological indicators potentially associated with stress regulation included cortisol, resting heart rate, resting systolic and diastolic blood pressure, and heart rate variability indices (RMSSD, SDNN, and LF/HF). These physiological measures may reflect autonomic, cardiovascular, or neuroendocrine processes but are not specific biomarkers of psychological stress. Each outcome was analyzed separately within its conceptual domain, without pooling across outcome categories. Resting physiological values were not interpreted as direct measures of stressor-evoked reactivity or recovery.

### 2.7 Statistical analysis

All statistical analyses were performed using R version 4.3.2, primarily with the metafor package (Viechtbauer, 2010). Given that different studies used different measurement instruments or units, continuous outcomes were analyzed by calculating Hedges’ g and its 95% confidence interval (95% CI) from the post-intervention means, standard deviations, and sample sizes of the intervention and control groups, with correction for small-sample bias. Effect sizes were calculated as intervention minus control: negative g indicated lower anxiety, depressive symptoms, perceived/general stress, fatigue, resting systolic/diastolic blood pressure, resting heart rate, blood cortisol, or LF/HF, whereas positive g indicated higher RMSSD or SDNN. Lower symptom scores and higher RMSSD/SDNN were interpreted as potentially favorable, while decreases in resting cardiovascular measures, cortisol, or LF/HF were interpreted contextually and did not alone establish improved stress regulation. For outcomes reported by at least three independent studies, pooled estimates were obtained using an inverse-variance-weighted random-effects model, with between-study variance (τ^2^) estimated using restricted maximum likelihood (REML), and 95% CIs and *p* values calculated using the normal-distribution-based *Z* test in the primary analysis. Between-study heterogeneity was assessed using Cochran’s Q, τ^2^, and *I*^2^ statistics (Higgins et al., 2003). For cluster-randomized trials that did not report intracluster correlation coefficients or results adjusted for clustering, the reported post-intervention group-level data were used without additional adjustment for the design effect, and the resulting uncertainty was considered in the risk-of-bias and GRADE assessments. The robustness of the results was examined using an *ad hoc*-adjusted Knapp–Hartung method, the DerSimonian–Laird estimator, analyses restricted to randomized trials, restriction analyses addressing assumptions regarding data transformations, and leave-one-out analyses; in the leave-one-out analyses, τ^2^ was re-estimated after each study was sequentially omitted (DerSimonian and Laird, 1986; Knapp and Hartung, 2003). When an outcome included ≥10 independent studies, potential small-study effects were assessed using funnel plots and Egger’s weighted regression test with standard error as the predictor, with *p* < 0.10 considered suggestive evidence (Egger et al., 1997; Sterne et al., 2011). Except for Egger’s test, all statistical tests were two-sided, and *p* < 0.05 was considered statistically significant. Effect sizes, confidence intervals, heterogeneity, sensitivity analyses, and certainty of evidence jointly informed interpretation.

## 3 Results

### 3.1 Study selection and characteristics

The database searches identified 6,646 records before deduplication: PubMed (*n* = 2,204), Web of Science (*n* = 1,951), Embase (*n* = 1,500), and the Cochrane Library (*n* = 991). Database-specific search yields are summarized in Supplementary Table S2. After deduplication, 4,315 records underwent title and abstract screening, 101 reports were assessed in full text, and 66 reports were excluded with reasons. The study selection process is shown in Figure 1.

**Figure 1:**
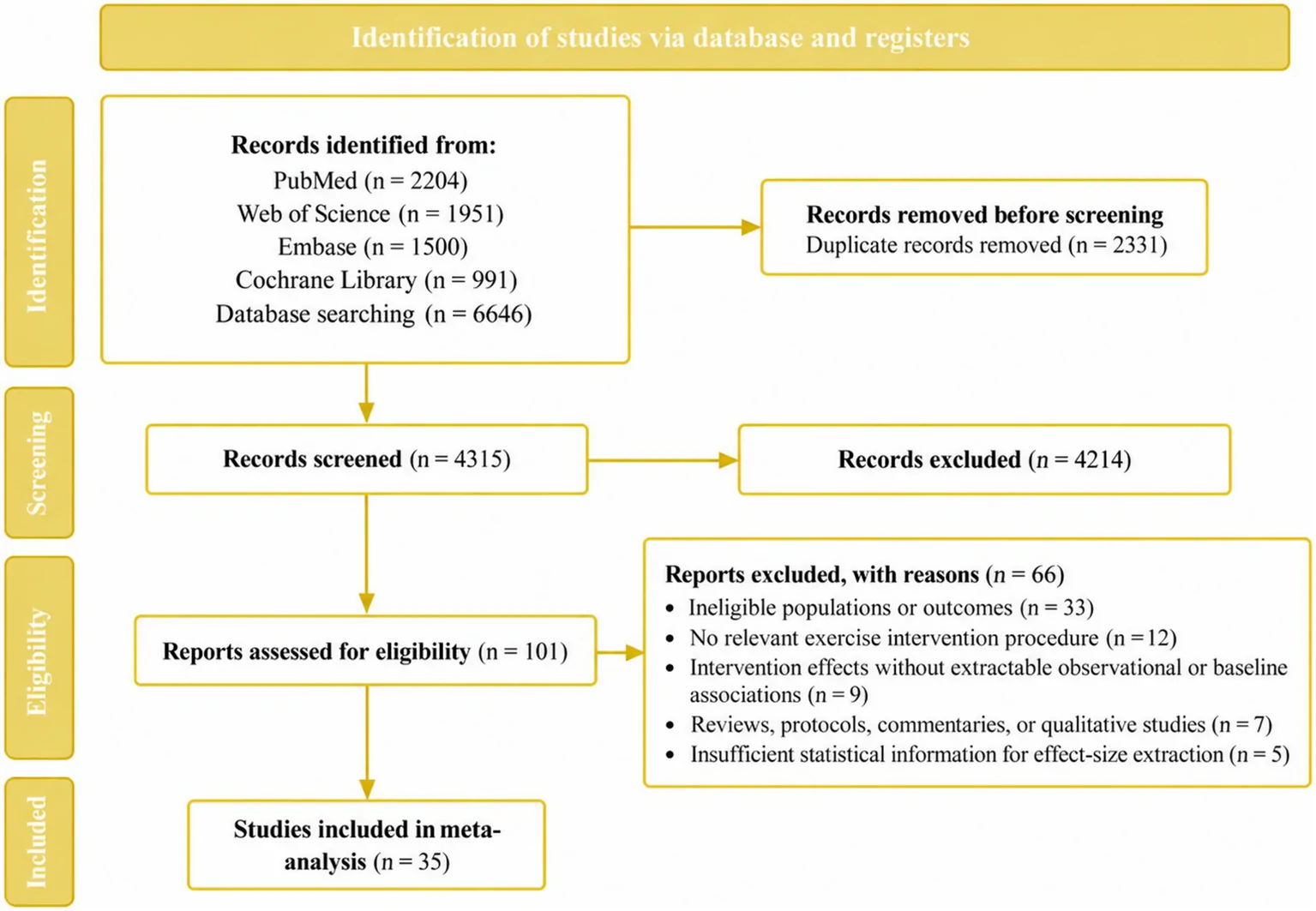
PRISMA flow diagram of the study selection process.

The quantitative synthesis included 35 unique reports. The Khalsa et al., 2012 report was coded as two outcome-specific entries, yielding 36 study labels in the analytical dataset. It contributed no more than one effect estimate to any single outcome. Of the 35 reports, 32 were randomized studies, comprising 20 individually randomized trials and 12 cluster-randomized trials. Three reports described non-randomized intervention studies.

Participants were approximately 8–19 years old and were recruited mainly from schools. Several studies included young athletes or participants with a defined training background. The reports covered 18 countries or regions. Interventions lasted from 3 weeks to 10 months and included aerobic exercise, high-intensity interval training, resistance training, sport-based training, yoga, qigong, and multicomponent school programs. Comparators included usual physical education, usual activity or training, attention or health education controls, and no-intervention or wait-list conditions.

Eleven outcomes met the quantitative synthesis threshold: anxiety, depressive symptoms, perceived or general stress, fatigue, resting systolic blood pressure, resting diastolic blood pressure, resting heart rate, blood cortisol, and the heart rate variability indices LF/HF, RMSSD, and SDNN. The outcome-specific analyses contained 4–15 studies and 90–3,157 participants. Study characteristics are presented in Supplementary Table S3. School-based yoga and mindfulness programs contributed anxiety outcomes (Khalsa et al., 2012; Quach et al., 2016; Bazzano et al., 2022; Arslan and Ardic, 2026). Anxiety was also assessed in qigong and aerobic exercise trials (Norris et al., 1992; Crews et al., 2004; Chang et al., 2013; Akko et al., 2020). Boxing, yoga, HIIT, and qigong studies provided further anxiety data (Rodrigues et al., 2021; Cioffi and Lubetzky, 2023; Mona et al., 2024; Ouerghi et al., 2026). Additional school-based and pilot physical activity trials also measured anxiety (Philippot et al., 2019; Parajuli et al., 2022; da Silva et al., 2025). Depressive symptoms were reported in school-based yoga and other physical activity trials (Noggle et al., 2012; Pant et al., 2013; Richards et al., 2014; Butzer et al., 2017). Ranjani et al. (2023) contributed perceived-stress data, while Sarabia et al. (2015) reported fatigue. Resting blood pressure or heart rate was assessed in school and laboratory-based physical activity trials (Hagins et al., 2013; Yu et al., 2016; Larsen et al., 2020; Ketelhut et al., 2021). Further cardiovascular outcomes came from qigong, HIIT, and structured physical-activity interventions (Sharma et al., 2017; Meng et al., 2022; Ketelhut et al., 2024; Domaradzki et al., 2025). HRV outcomes were reported after interval running, soccer, yoga or mindfulness, and school-based HIIT (Ketelhut et al., 2024; Gamelin et al., 2009; Hammami et al., 2017; Ivaki et al., 2021). Blood cortisol was assessed in resistance-training, aerobic-training, and HIIT studies (Ouerghi et al., 2026; Gorostiaga et al., 1999; Jeon and Ha, 2015; Darragi et al., 2026).

### 3.2 Risk of bias

Risk of bias was assessed for 36 analytical entries representing 35 unique reports. Thirty-three randomized entries were assessed using RoB 2, whereas three non-randomized intervention studies were assessed using ROBINS-I. The domain-level summary is presented in Supplementary Figure S1, and the study-level RoB 2 and ROBINS-I judgments are shown in Supplementary Figures S2 and S3, respectively.

Among the RoB 2 assessments, 8 entries (24.2%) were judged to have a high overall risk of bias, and 25 (75.8%) raised some concerns. None were judged to have a low overall risk of bias. The two Khalsa entries originated from the same report. After consolidation, 7 of 32 randomized reports were classified as high risk, while 25 raised some concerns.

Concerns regarding the randomization process were identified in 25 entries (75.8%), whereas 8 entries (24.2%) were judged to have low risk. All randomized entries raised some concerns regarding deviations from intended interventions and selection of the reported result. No entries were rated high risk in these two domains or in the randomization domain (0/33 in each). Outcome measurement was judged to have a high risk of bias in 7 entries (21.2%), some concerns in 13 (39.4%), and low risk in 13 (39.4%). Missing outcome data were judged to have a high risk of bias in 1 entry (3.0%), some concerns in 25 (75.8%), and low risk in 7 (21.2%). All 13 cluster-randomized entries raised some concerns regarding participant identification and recruitment, with none rated high risk (0/13). The main concerns involved insufficient detail to establish allocation concealment, appropriate handling of intervention deviations and analysis populations, and prespecification of analyses. High-risk judgments for outcome measurement mainly reflected unblinded participant-reported psychological outcomes. Incomplete reporting limited definitive judgments about intervention deviations and selective reporting.

All three non-randomized studies assessed using ROBINS-I were judged to have a serious overall risk of bias. Confounding was rated as serious in all three studies (100%). Participant selection was rated as serious in two studies (66.7%) and moderate in one (33.3%). Deviations from intended interventions were rated as moderate in all three studies. Missing data were rated as serious in one study (33.3%) and moderate in two (66.7%). Outcome measurement was rated as low risk in two studies and moderate risk in one. The reporting domain was rated as moderate in all three studies. Classification of interventions was rated low risk in all three studies; no serious or critical ratings were assigned for intervention classification, deviations from intended interventions, outcome measurement, or selection of the reported result (0/3 in each). Across the evidence base, these limitations reduced confidence in the pooled estimates. Expectations in unblinded self-reports could exaggerate apparent benefit, whereas missing data and confounding could shift estimates in either direction; the net direction and magnitude of bias could not be established.

### 3.3 Results of data synthesis

Pooled effect estimates and their 95% prediction intervals are presented in Supplementary Table S4.

#### 3.3.1 Effects of physical activity interventions on anxiety in children and adolescents

A total of 15 studies evaluated the effects of physical activity interventions on anxiety levels in children and adolescents (Figure 2). The meta-analysis showed that, compared with the control group, anxiety levels tended to be lower in the intervention group; however, the between-group difference did not reach statistical significance (Hedges’ *g* = −0.34, 95% CI: −0.79 to 0.11, *p* = 0.137). Negative values denote lower post-intervention anxiety scores in the intervention groups.

**Figure 2:**
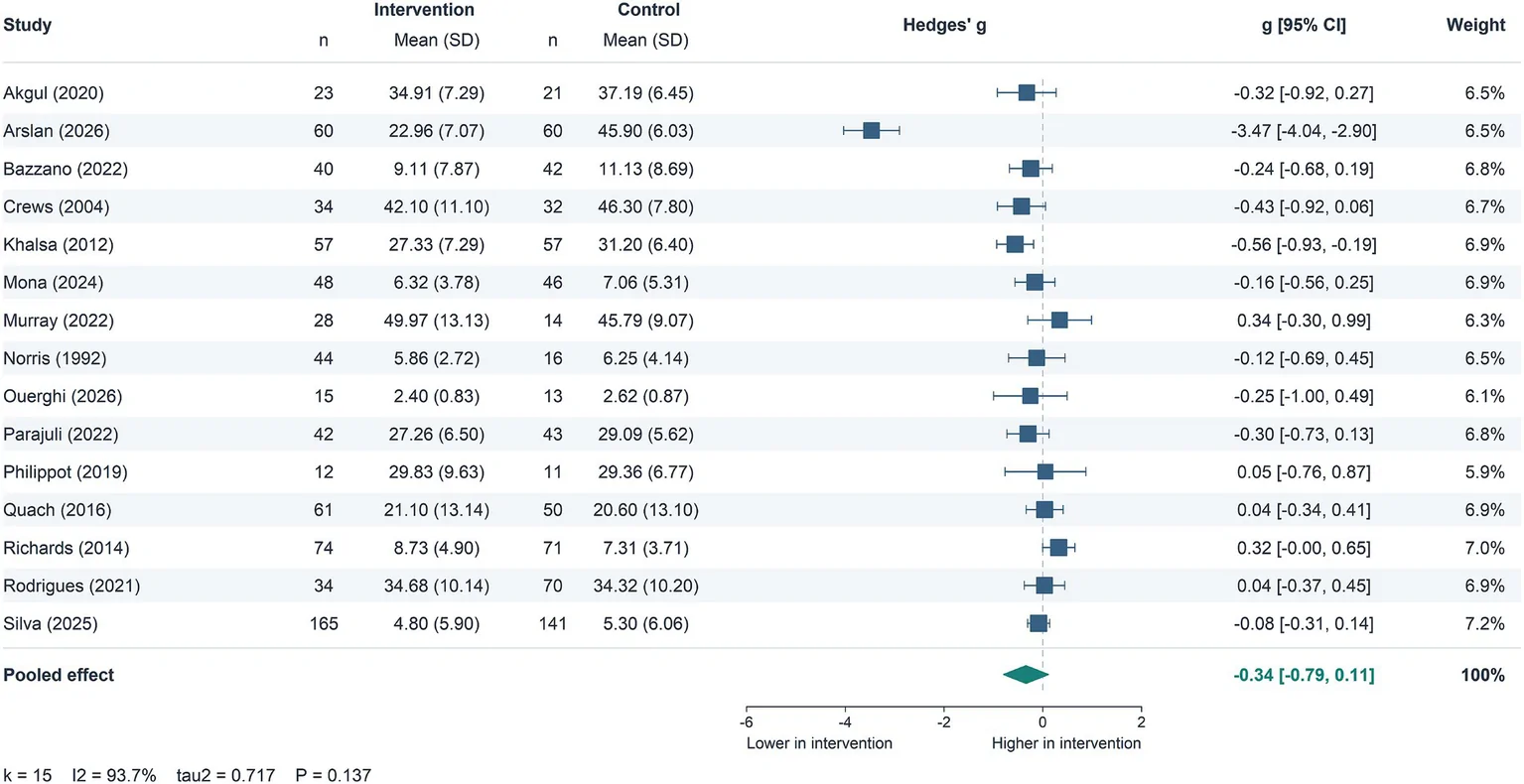
Effects of physical activity interventions on anxiety in children and adolescents.

Considerable heterogeneity was observed across studies (*I*^2^ = 93.7%, τ^2^ = 0.717), indicating substantial variation in effect estimates among individual studies. The findings of individual studies also showed some inconsistency, with the study by Khalsa et al. (2012) reporting a statistically significant difference favoring the intervention group, whereas several other studies reported effect sizes close to zero or slight effects in the opposite direction.

#### 3.3.2 Effects of physical activity interventions on depressive symptoms in children and adolescents

Thirteen studies were included to evaluate the effects of physical activity interventions on depressive symptoms in children and adolescents (Figure 3). The meta-analysis showed that, compared with the control group, the primary pooled estimate favored lower depressive symptom scores and was statistically significant (Hedges’ g = −0.53, 95% CI: −1.04 to −0.02, *p* = 0.041).

**Figure 3:**
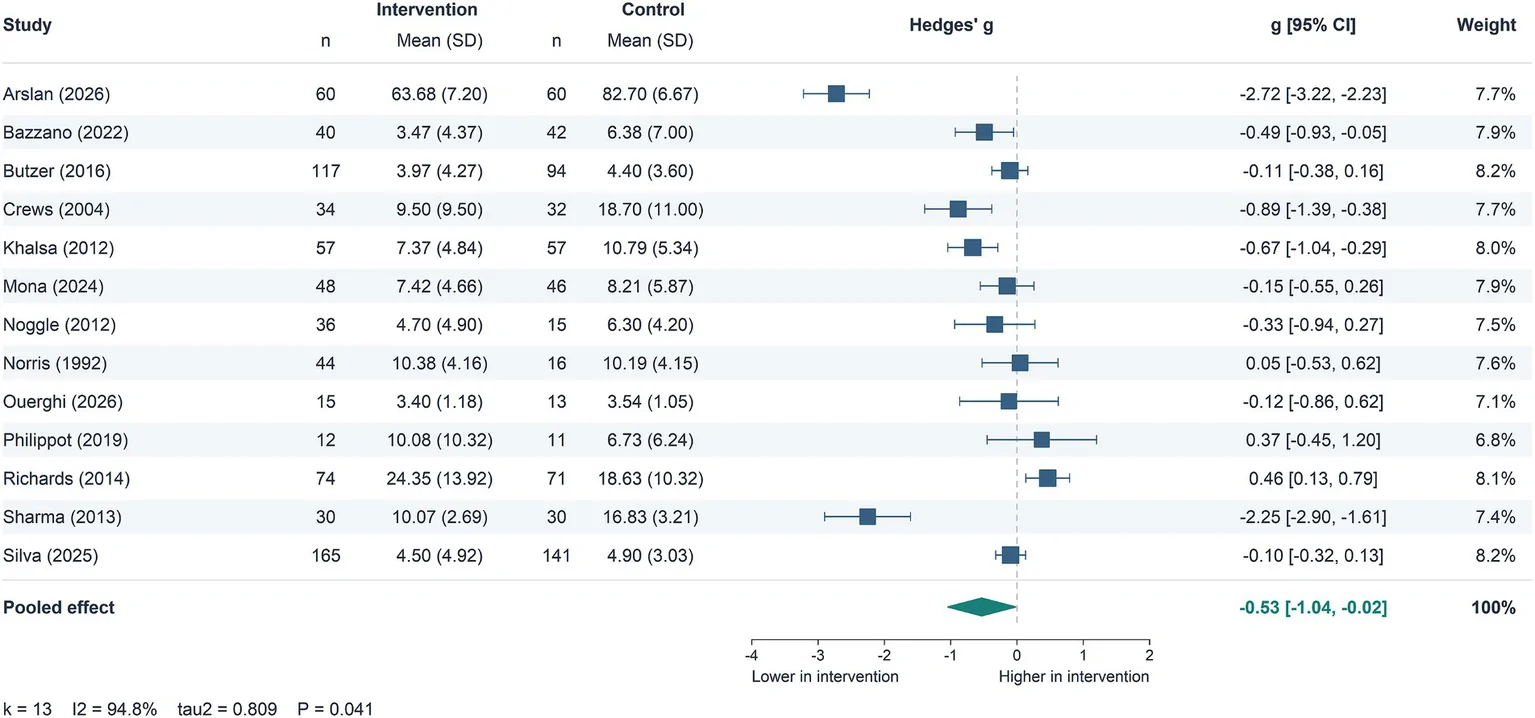
Effects of physical activity interventions on depressive symptoms in children and adolescents.

Substantial heterogeneity was observed across studies (*I*^2^ = 94.8%, τ^2^ = 0.809), indicating considerable variation in intervention effects across individual studies. The individual study estimates showed that most studies favored the intervention, with lower levels of depressive symptoms observed in the intervention groups; however, the magnitude of the effects varied considerably across studies, and the 95% CIs of some studies crossed the line of no effect.

#### 3.3.3 Effects of physical activity interventions on perceived/general stress in children and adolescents

Seven studies were included to evaluate the effects of physical activity interventions on perceived/general stress in children and adolescents (Figure 4). The meta-analysis showed that, compared with the control group, the primary pooled estimate favored lower perceived/general stress and was statistically significant (Hedges’ g = −0.28, 95% CI: −0.48 to −0.08, *p* = 0.006). Moderate heterogeneity was observed across studies (*I*^2^ = 49.9%, τ^2^ = 0.030), indicating variation in effect sizes.

**Figure 4:**
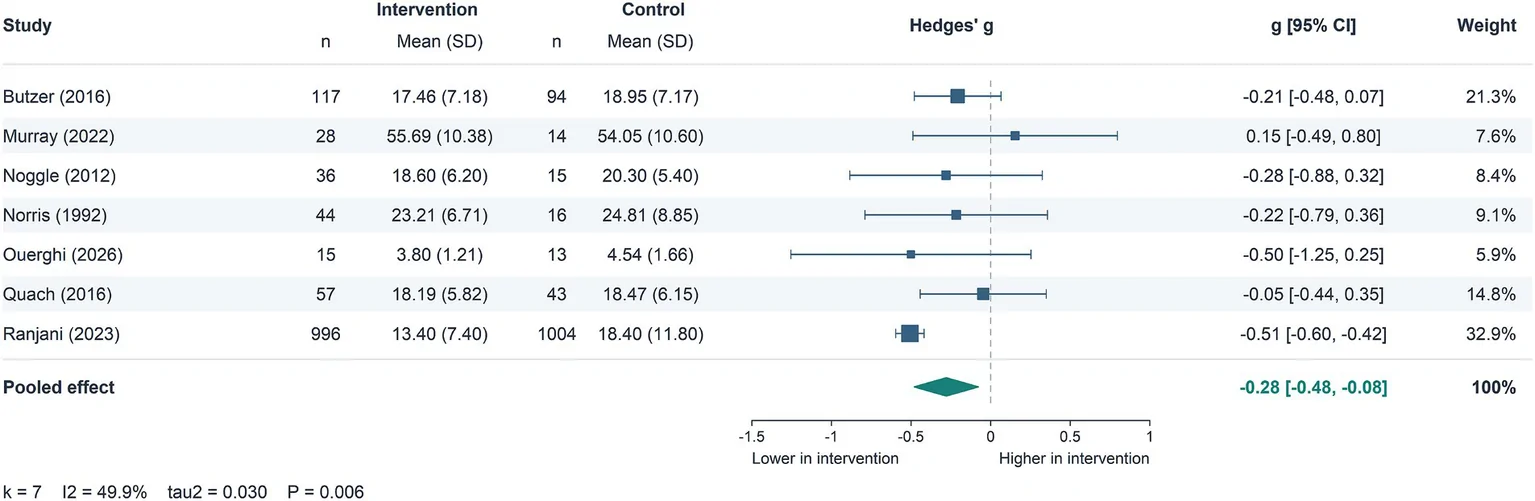
Effects of physical activity interventions on perceived / general stress in children and adolescents.

#### 3.3.4 Effects of physical activity interventions on fatigue in children and adolescents

Four studies evaluated the effects of physical activity interventions on fatigue in children and adolescents (Figure 5). The meta-analysis showed that, compared with the control group, the pooled point estimate favored lower fatigue, although the between-group difference did not reach statistical significance (Hedges’ g = −0.11, 95% CI: −0.32 to 0.10, *p* > 0.05).

**Figure 5:**
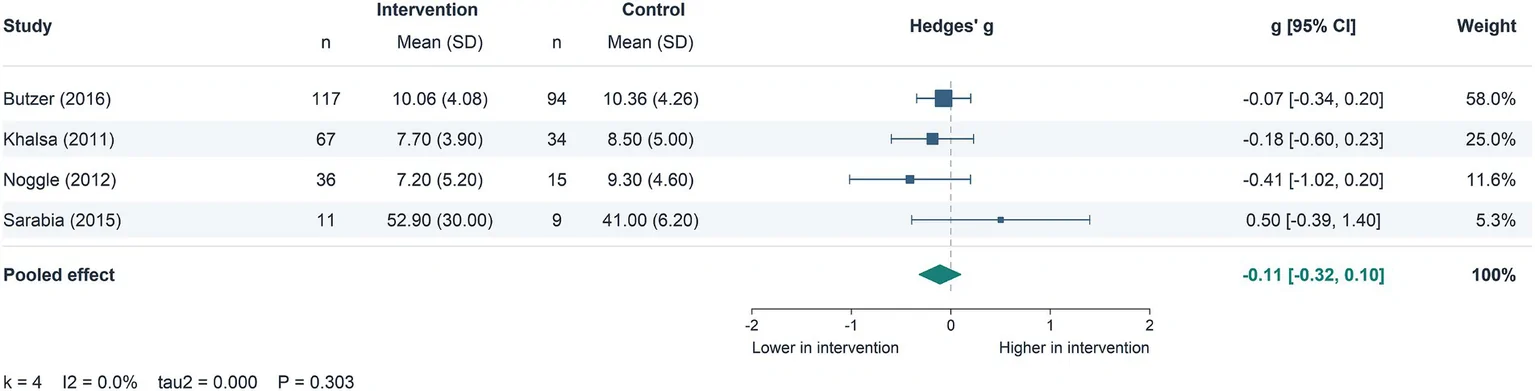
Effects of physical activity interventions on fatigue in children and adolescents.

Statistical heterogeneity was estimated as zero (*I*^2^ = 0.0%, τ^2^ = 0.000). With only four studies, however, these estimates should be interpreted cautiously and do not establish that the study effects were homogeneous. Although some variation was observed in the direction and magnitude of the effects across individual studies, the overall effect estimate was close to the null effect, and the 95% CI crossed the line of no effect. Therefore, given the limited number of available studies, the potential effects of physical activity interventions on fatigue in children and adolescents should be interpreted with caution, and further high-quality studies are warranted to verify these findings.

#### 3.3.5 Effects of physical activity interventions on resting systolic blood pressure in children and adolescents

Ten studies were included to evaluate the effects of physical activity interventions on resting systolic blood pressure in children and adolescents (Figure 6). The meta-analysis showed that, compared with the control group, the pooled estimate favored lower resting systolic blood pressure and was statistically significant (Hedges’ *g* = −0.15, 95% CI: −0.26 to −0.04, *p* = 0.008). The negative effect size indicates that physical activity interventions were generally associated with lower resting systolic blood pressure in children and adolescents. Although the pooled effect size was small, its 95% CI did not cross the null value, indicating that the observed difference was statistically significant.

**Figure 6:**
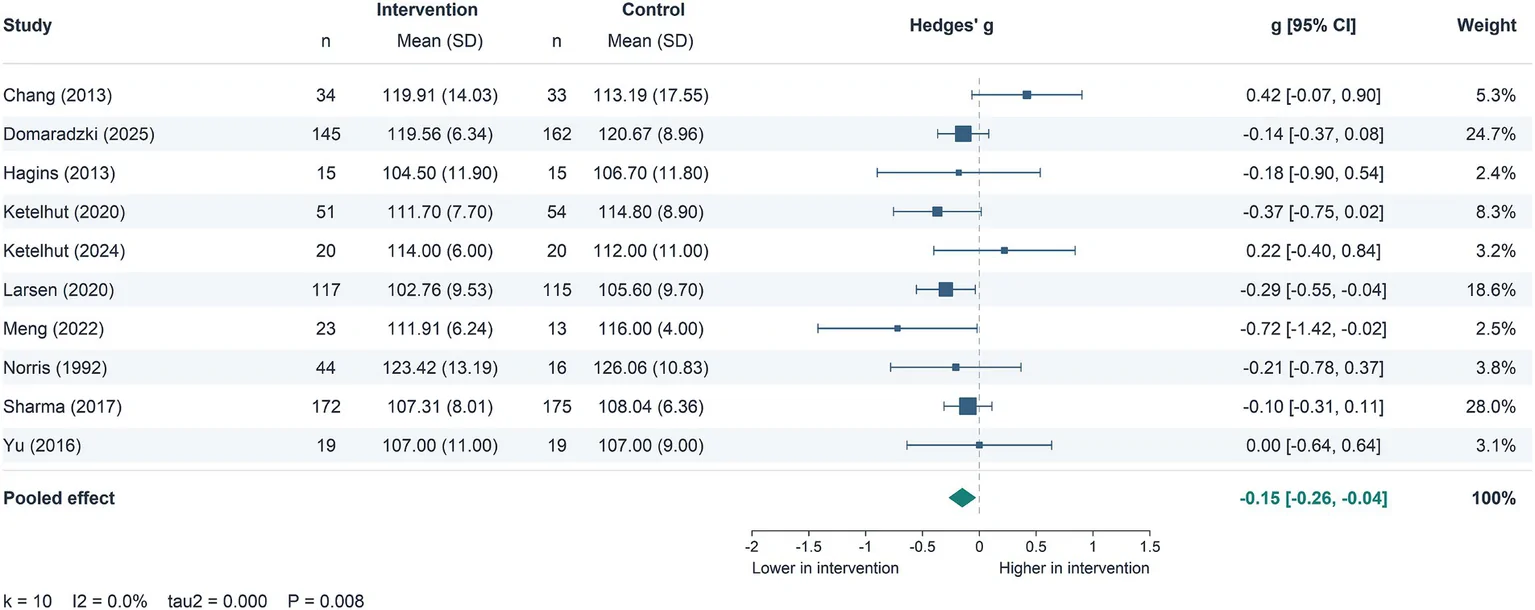
Effects of physical activity interventions on resting systolic blood pressure in children and adolescents.

Statistical heterogeneity was estimated as zero (*I*^2^ = 0.0%, τ^2^ = 0.000), although these estimates do not exclude clinically or methodologically meaningful differences between studies. Across the individual studies, the effect estimates generally favored lower resting systolic blood pressure in the intervention groups.

#### 3.3.6 Effects of physical activity interventions on resting diastolic blood pressure in children and adolescents

A total of 10 studies evaluated the effects of physical activity interventions on resting diastolic blood pressure in children and adolescents (Figure 7). The meta-analysis showed that, compared with the control group, the primary pooled estimate favored lower resting diastolic blood pressure and was statistically significant (Hedges’ g = −0.33, 95% CI: −0.64 to −0.01, *p* = 0.041). The negative effect size indicates that physical activity interventions were overall associated with lower resting diastolic blood pressure in children and adolescents. Although the pooled effect size was small, its 95% CI did not cross the null value, indicating that the observed difference was statistically significant.

**Figure 7:**
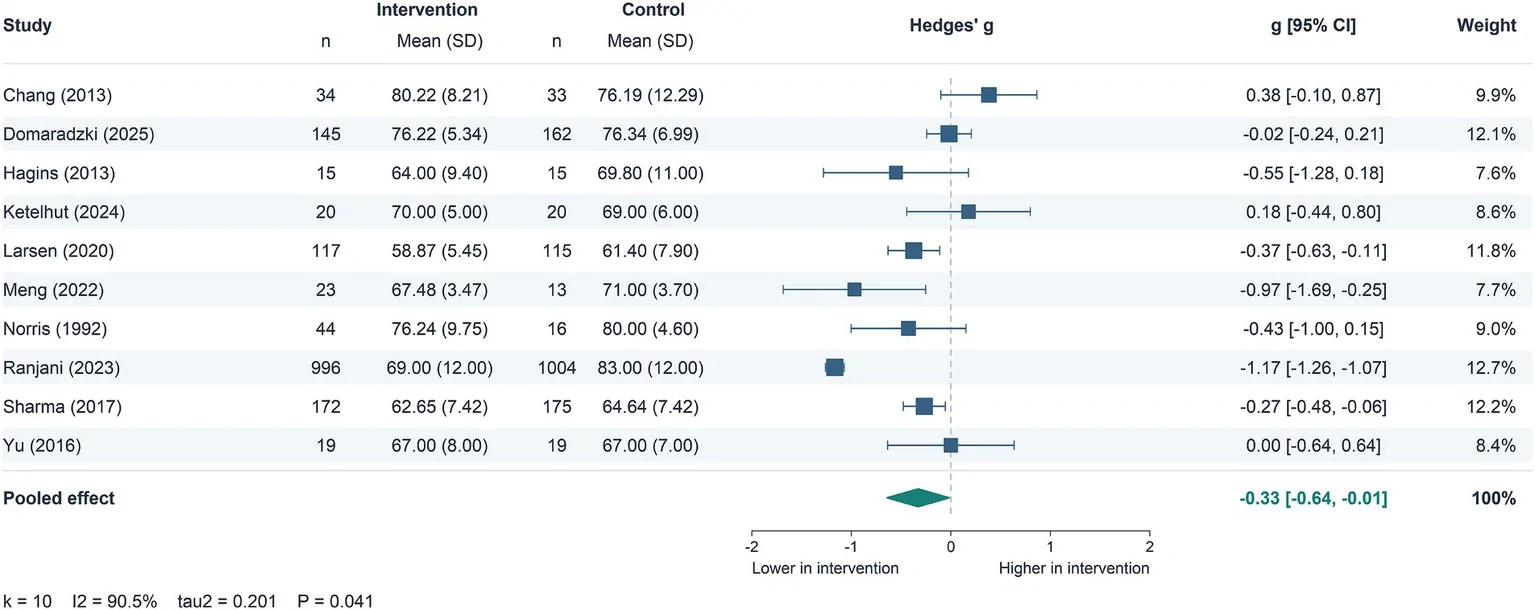
Effects of physical activity interventions on resting diastolic blood pressure in children and adolescents.

Considerable heterogeneity was observed across studies (*I*^2^ = 90.5%, τ^2^ = 0.201), indicating substantial variation in the magnitude of intervention effects across studies.

#### 3.3.7 Effects of physical activity interventions on blood cortisol in children and adolescents

Four studies evaluated the effects of physical activity interventions on blood cortisol levels in children and adolescents (Figure 8). The meta-analysis showed that physical activity interventions did not have a statistically significant effect on blood cortisol levels in children and adolescents (Hedges’ g = −0.07, 95% CI: −0.80 to 0.67, *p* > 0.05). Furthermore, substantial heterogeneity was observed among the included studies (*I*^2^ = 66.3%, τ^2^ = 0.373); therefore, given the limited number of available studies, the potential effects of physical activity interventions on blood cortisol levels in children and adolescents should be interpreted cautiously, and further high-quality studies are warranted to confirm these findings.

**Figure 8:**
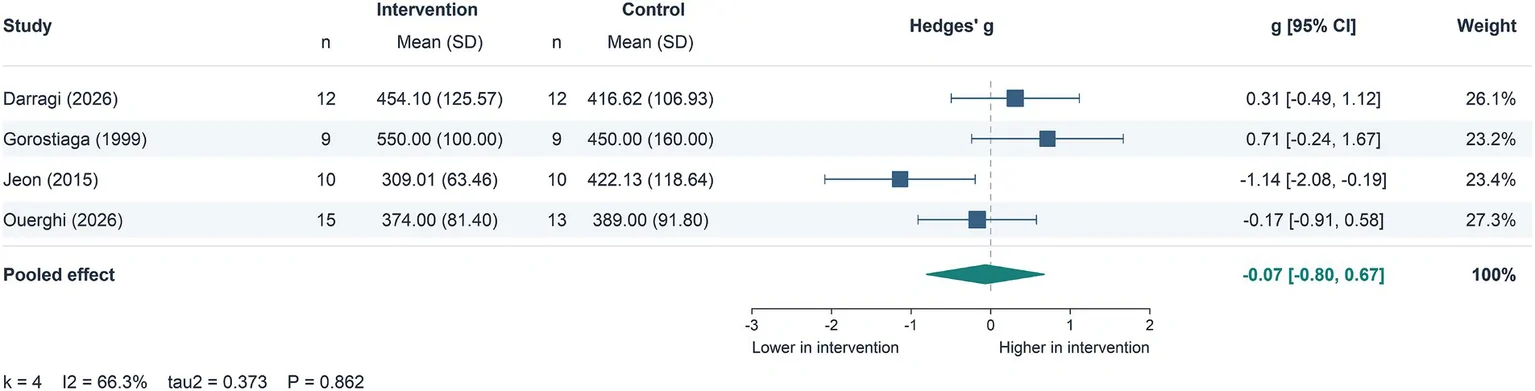
Effects of physical activity interventions on blood cortisol in children and adolescents.

#### 3.3.8 Effects of physical activity interventions on HRV in children and adolescents

Four studies assessed each of HRV-RMSSD, HRV-SDNN, and HRV-LF/HF (Figure 9, Figure 10, and Figure 11). The meta-analysis showed that physical activity interventions had no statistically significant effects on HRV-RMSSD (Hedges’ g = 0.03, 95% CI: −0.31 to 0.36, *p* > 0.05, I^2^ = 0.0%, *τ*^2^ = 0.000) or HRV-SDNN (Hedges’ g = 0.01, 95% CI: −0.32 to 0.35, *p* > 0.05, *I*^2^ = 0.0%, τ^2^ = 0.000) in children and adolescents. Although *I*^2^ and τ^2^ were estimated as zero for both indices, only four studies contributed to each analysis, limiting the assessment of heterogeneity. Although some variation was observed in the direction and magnitude of effects across individual studies, the overall effect estimates were close to the line of no effect, and the 95% CIs crossed the null value. Therefore, given the limited number of available studies, the potential effects of physical activity interventions on HRV-RMSSD and HRV-SDNN in children and adolescents should be interpreted with caution. For HRV-LF/HF, the primary pooled estimate favored lower LF/HF and was statistically significant (Hedges’ *g* = −0.43, 95% CI: −0.80 to −0.05, *p* = 0.028, *I*^2^ = 29.2%, τ^2^ = 0.044).

**Figure 9:**
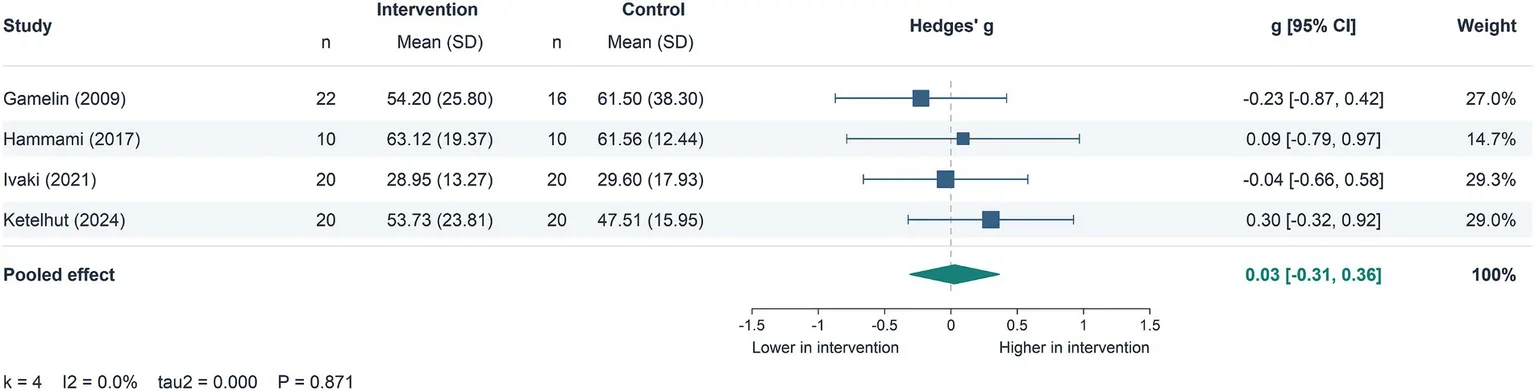
Effects of physical activity interventions on HRV-RMSSD in children and adolescents.

**Figure 10:**
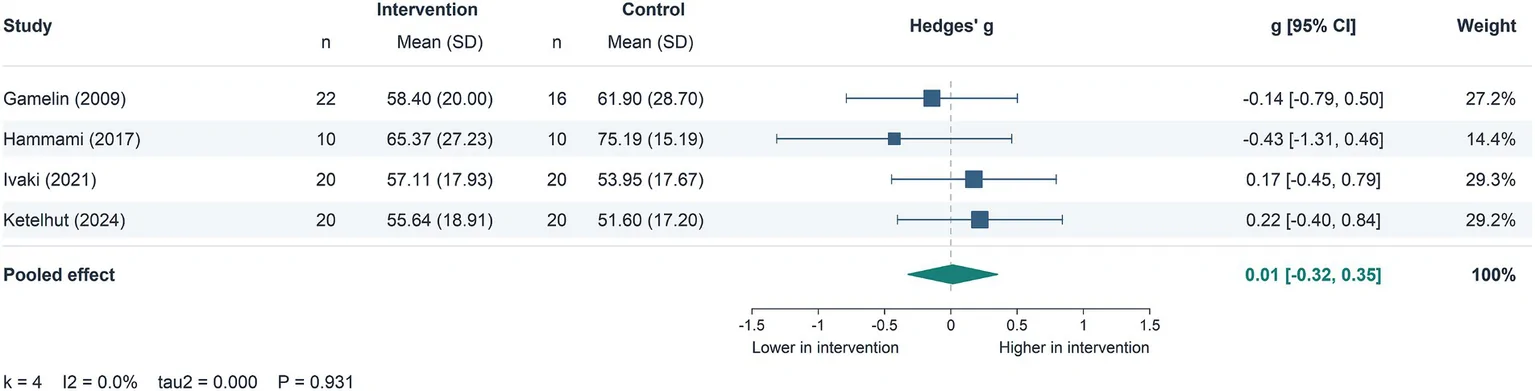
Effects of physical activity interventions on HRV-SDNN in children and adolescents.

**Figure 11:**
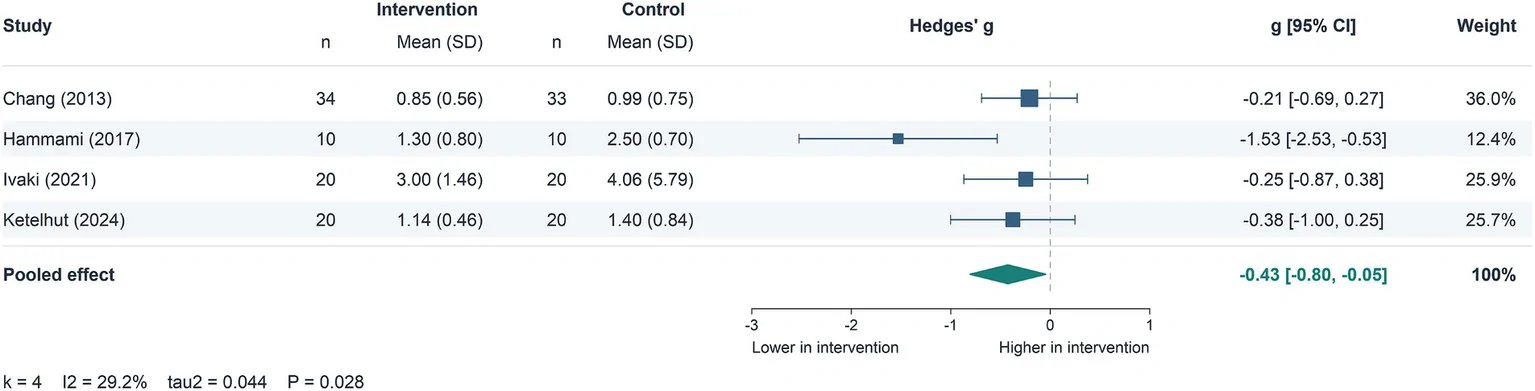
Effects of physical activity interventions on HRV-LF/HF in children and adolescents.

### 3.4 Sensitivity analyses

The robustness of the pooled estimates was examined using alternative random-effects estimators and leave-one-out analyses. Under modified Hartung–Knapp inference, the effects for perceived or general stress and systolic blood pressure remained statistically significant. The confidence intervals for depressive symptoms, diastolic blood pressure, and LF/HF crossed the null (Supplementary Table S5). With the DerSimonian–Laird estimator and normal inference, depressive symptoms, perceived or general stress, systolic blood pressure, and LF/HF remained statistically significant, whereas diastolic blood pressure did not.

Leave-one-out analyses did not change the statistical significance of anxiety, fatigue, resting heart rate, blood cortisol, RMSSD, or SDNN. The depressive symptom estimate became non-significant after omitting Arslan and Ardic, 2026; Bazzano et al., 2022; Crews et al., 2004; Khalsa et al., 2012; Noggle et al., 2012; Pant et al., 2013. The perceived stress estimate became non-significant only after omitting Ranjani et al. (2023). The systolic blood pressure estimate became non-significant after omitting Domaradzki et al., 2025; Larsen et al., 2020; Sharma et al., 2017. The diastolic blood pressure estimate became non-significant after omitting Hagins et al., 2013; Larsen et al., 2020; Meng et al., 2022; Norris et al., 1992; Ranjani et al., 2023; Sharma et al., 2017. The LF/HF estimate became non-significant after omitting any one of its four contributing studies.

Excluding the three non-randomized studies preserved the significant effects for depressive symptoms, perceived or general stress, and systolic blood pressure. The effects for diastolic blood pressure and LF/HF were no longer statistically significant after this restriction. These findings indicated that several pooled estimates were sensitive to model specification and the contribution of individual studies. Leave-one-out sensitivity-analysis results are provided in Supplementary Table S6.

### 3.5 Certainty of evidence

The certainty of evidence ranged from low to very low. Low certainty was assigned to resting systolic blood pressure, fatigue, and resting heart rate. Very low certainty was assigned to anxiety, depressive symptoms, perceived or general stress, resting diastolic blood pressure, blood cortisol, RMSSD, SDNN, and LF/HF. No outcome received a high or moderate-certainty rating.

Evidence for systolic blood pressure was rated as low certainty following downgrades for risk of bias and imprecision. Its 95% CI (−0.26 to −0.04) included reductions both smaller and larger than the 0.20-SD benchmark. The fatigue (−0.32 to 0.10; *n* = 383) and resting heart rate (−0.28 to 0.08; *n* = 487) intervals included both a reduction exceeding this benchmark and little or no difference; each outcome was downgraded one level for imprecision. Their nominal sample sizes were also below the approximate information-size benchmark of 786 participants. For systolic blood pressure, leave-one-out point estimates remained negative (−0.182 to −0.117), although three intervals crossed zero. These leave-one-out results indicated statistical fragility despite a consistent direction of effect; no additional downgrade was applied. Anxiety and depressive symptoms were downgraded for risk of bias, very serious inconsistency, and imprecision; perceived or general stress was downgraded for risk of bias, inconsistency, and imprecision.

Diastolic blood pressure was downgraded for risk of bias, very serious inconsistency, imprecision, and suspected small-study effects. Blood cortisol was downgraded for risk of bias, inconsistency, indirectness, and very serious imprecision. RMSSD and SDNN were downgraded for risk of bias, indirectness, and very serious imprecision; their intervals crossed both standardized magnitude thresholds. LF/HF was downgraded for risk of bias, indirectness, and imprecision. Sensitivity-analysis findings informed the interpretation of domain-specific judgments without an additional downgrade. Supplementary Table S1 provides the operational criteria, information-size assumptions, and explanations for each downgrade. The imprecision judgments, and hence the certainty ratings, are conditional on this distribution-based benchmark and do not establish whether effects are clinically important for each outcome.

### 3.6 Publication bias

Egger’s regression was conducted for the four outcomes represented by at least 10 studies (Egger et al., 1997). The tests were non-significant for anxiety (*k* = 15, *t* = −1.00, *p* = 0.337) and depressive symptoms (*k* = 13, *t* = −1.34, *p* = 0.208). The test was also non-significant for resting systolic blood pressure (*k* = 10, *t* = 0.22, *p* = 0.830). In contrast, the result for resting diastolic blood pressure was statistically significant (*k* = 10, *t* = 2.54, *p* = 0.034), indicating potential small-study effects, for which publication bias remains a plausible explanation. Egger’s regression was not performed for the remaining outcomes because fewer than 10 studies were available and the power to detect asymmetry would be limited. Publication bias cannot be excluded for these outcomes or for those with non-significant test results. Funnel-plot asymmetry may also arise from between-study heterogeneity or associations between effect sizes and their standard errors (Sterne et al., 2011). The relative contributions of heterogeneity and publication bias to the observed asymmetry could not be determined. The predominance of estimates favoring the intervention for several psychological and cardiovascular outcomes is compatible with selective publication of favorable studies or non-reporting of unfavorable outcomes, although effect direction alone cannot establish either form of bias. Underrepresentation of null or unfavorable findings could overestimate the apparent benefits. Detailed test results are reported in Supplementary Table S7; the corresponding funnel plots for anxiety, depressive symptoms, resting systolic blood pressure, and resting diastolic blood pressure are shown in Supplementary Figures S4–S7, respectively.

## 4 Discussion

This systematic review and meta-analysis aimed to evaluate the effects of physical activity interventions on stress-related psychological and physiological outcomes in children and adolescents and to comprehensively synthesize the existing evidence across both physiological and psychological dimensions. Overall, the findings suggested possible differences in selected stress-related outcomes, with certainty varying by outcome. For psychological outcomes, very-low-certainty evidence suggested possible reductions in perceived/general stress and depressive symptoms, whereas the effects on anxiety and fatigue remained uncertain. For physiological outcomes, low-certainty evidence suggested a possible small reduction in resting systolic blood pressure, while very-low-certainty evidence suggested possible reductions in resting diastolic blood pressure and HRV-LF/HF. No statistically significant differences were observed for resting heart rate, blood cortisol, HRV-RMSSD, or HRV-SDNN. Notably, the effects of physical activity interventions were not entirely consistent across outcomes, and substantial heterogeneity was observed for some outcomes. These findings suggest that intervention effects differ across outcomes, without establishing a common improvement in stress regulation, and that the magnitude of these effects may be jointly influenced by factors such as activity type, intervention intensity and duration, participant characteristics, and outcome measurement methods (Campbell and Ehlert, 2012; Mücke et al., 2018).

### 4.1 Effects of physical activity interventions on psychological outcomes in children and adolescents

Very-low-certainty evidence suggested possible reductions in perceived/general stress and depressive symptoms. The depressive symptom finding was particularly uncertain because of very high heterogeneity, a confidence interval close to the null boundary, and sensitivity to the inferential method and individual studies. Recchia et al. reported a smaller depressive symptom effect and less heterogeneity (*g* = −0.29; *I*^2^ = 62.9%) than the present synthesis (*g* = −0.53; *I*^2^ = 94.8%), although the confidence intervals overlapped (Recchia et al., 2023). The conceptual distinction between depressive symptoms and perceived stress limits the implications of this finding for stress regulation. This finding is broadly consistent with previous research examining the relationship between physical activity and psychological health in children and adolescents (Biddle et al., 2019; Carter et al., 2016). Physical activity may help children and adolescents cope more effectively with daily stress through mechanisms such as promoting positive emotions, enhancing self-efficacy, improving emotional regulation, and increasing social interaction (Lubans et al., 2016). In addition, possible changes in perceived stress may be partly related to the effects of physical activity on autonomic nervous and neuroendocrine function, suggesting that the psychological effects of physical activity may result from the combined influence of physiological adaptation, emotional regulation, and changes in social behavior (Pascoe et al., 2020). Notably, the estimated reduction in depressive symptoms was relatively greater, whereas the effect size for perceived stress was smaller, suggesting that the effects of physical activity may vary across psychological outcomes and may be influenced not only by the activity itself but also by individual psychological characteristics and environmental factors.

Notably, this study found no statistically significant effects of physical activity interventions on anxiety or fatigue. With effects expressed in the same direction, Andermo et al.’s anxiety estimate (*g* = −0.35; *I*^2^ = 64%) was similar in magnitude but less heterogeneous than the present estimate (*g* = −0.34; *I*^2^ = 93.7%) (Andermo et al., 2020). Different eligibility criteria and control conditions may partly explain these discrepancies, including the broader age range (up to 25 years) in Carter et al.’s review (Carter et al., 2021). This finding suggests that the effects of physical activity interventions on anxiety may not be universal but may depend substantially on individual characteristics and the intervention context (Carter et al., 2021). Compared with perceived stress, anxiety reflects not only an individual’s subjective appraisal of stressful events but also persistent emotional vulnerability, cognitive biases, and individual psychological characteristics; therefore, physical activity alone may be insufficient to produce stable and consistent improvements. In addition, the included studies varied in terms of activity type, intensity, frequency, and duration, as well as the instruments used to assess anxiety, and whether different activity doses can reach the threshold required to improve anxiety remains to be determined. The high degree of heterogeneity further suggests that the pooled effect may obscure potential differences in the true effects across populations and intervention protocols. In contrast to anxiety, fatigue is a distinctly multidimensional subjective experience that may arise from physical exertion and insufficient recovery and may also be influenced by sleep quality, academic demands, emotional states, and habitual activity levels. Accordingly, exercise may alleviate fatigue by improving physical fitness and sleep, while the additional physical load imposed by exercise may also temporarily increase perceived fatigue, with the ultimate effect potentially depending on the balance between exercise load and recovery capacity. Meanwhile, only a small number of studies assessed fatigue, and the limited sample size and statistical power may have reduced the ability to detect a true intervention effect. Therefore, the current evidence is more appropriately interpreted as insufficient to confirm that physical activity interventions significantly improve fatigue in children and adolescents, rather than as evidence that these interventions have no effect on fatigue.

Overall, the effects of physical activity on psychological outcomes in children and adolescents appear to be outcome-specific, with very-low-certainty evidence suggesting possible reductions in perceived stress and depressive symptom scores, whereas its effects on anxiety and fatigue remain uncertain (Biddle et al., 2019). Neill et al. identified only two studies assessing different stress domains and did not pool them, whereas the present seven-study synthesis provides a quantitative estimate for perceived/general stress, albeit with very low certainty (Neill et al., 2020). This variation suggests that future research should not evaluate the psychological effects of physical activity based solely on a single psychological indicator, but should instead adopt a multidimensional approach encompassing perceived stress, emotional states, and related behavioral outcomes, while further considering factors such as activity type, intensity, frequency, and intervention duration (Carter et al., 2021). Meanwhile, standardized psychological assessment tools should be used whenever possible, and subgroup or moderator analyses incorporating baseline psychological status and intervention adherence should be conducted to further clarify which intervention protocols, for which populations, and through which pathways may be more effective in improving stress-related psychological outcomes among children and adolescents.

### 4.2 Effects of physical activity interventions on physiological indicators in children and adolescents

The primary pooled estimates favored lower resting systolic blood pressure, resting diastolic blood pressure, and HRV-LF/HF, with low certainty for systolic blood pressure and very low certainty for the other two outcomes. Estimates for resting heart rate, blood cortisol, HRV-RMSSD, and HRV-SDNN did not reach statistical significance. These physiological outcomes have limited specificity for psychological stress (Campbell and Ehlert, 2012).

Regarding blood pressure, the certainty of evidence differed between systolic and diastolic outcomes, with low estimated statistical heterogeneity for systolic blood pressure but substantial heterogeneity and sensitivity to the inferential method for diastolic blood pressure. These findings suggest possible changes in resting cardiovascular measures among children and adolescents. Any such changes may reflect general cardiovascular training adaptations, including vascular and autonomic changes, rather than altered responses to psychological stress. The implications of lower resting blood pressure for stress regulation remain uncertain. As the participants included in this study were typically developing children and adolescents, these estimates describe possible changes in resting cardiovascular measures in this population and should not be directly extrapolated to the therapeutic effects of exercise in children with hypertension. Regarding HRV, the primary LF/HF estimate was statistically significant, but the evidence was of very low certainty. Statistical significance was lost after omission of any one of the four contributing studies, restriction to randomized studies, or use of modified Hartung–Knapp inference. The LF/HF finding was therefore statistically fragile despite relatively low observed heterogeneity. Estimates for RMSSD and SDNN were inconclusive. RMSSD mainly reflects short-term, vagally mediated variability, whereas SDNN summarizes overall variability over the recording period; neither directly measures psychological stress (Laborde et al., 2017; Task Force of the European Society of Cardiology and the North American Society of Pacing and Electrophysiology, 1996; Shaffer and Ginsberg, 2017). LF/HF is influenced by both sympathetic and parasympathetic activity, and its traditional interpretation as an index of “sympathovagal balance” has been increasingly questioned in recent years (Billman, 2013; Reyes del Paso et al., 2013). The observed LF/HF reduction therefore has uncertain implications for autonomic balance or psychological stress. Meanwhile, the absence of significant changes in RMSSD and SDNN further suggests that the effects of physical activity interventions on autonomic regulation may be complex and may be influenced by factors such as activity dose, measurement conditions, and intervention duration. In contrast, this study found no significant effects of physical activity interventions on resting heart rate or blood cortisol levels. As an important physiological indicator of hypothalamic–pituitary–adrenal (HPA) axis activity, cortisol levels are readily influenced by factors such as sampling time, acute stress, sleep, and individual circadian rhythms, which may contribute to variability in findings across studies (Adam and Kumari, 2009; Stalder et al., 2016). Resting heart rate also varies substantially with age (Fleming et al., 2011). Assessment of stress reactivity and recovery requires measurements during and after a defined stressor; resting heart rate and post-intervention cortisol concentrations characterize physiological state. Therefore, the current findings do not indicate that physical activity interventions have no effect on neuroendocrine or autonomic function in children and adolescents; rather, they suggest that the available evidence is currently insufficient to demonstrate consistent changes in these indicators.

Overall, the evidence for physiological stress-related indicators in children and adolescents varied in certainty and stability across outcomes. Reductions were observed in blood pressure and LF/HF, whereas no significant changes were observed in cortisol, resting heart rate, or certain HRV indices. Therefore, future research should use standardized physiological assessments and distinguish resting measurements from responses to a defined stressor and subsequent recovery (Laborde et al., 2017). In addition, factors such as activity type, intensity, frequency, and intervention duration should be further considered to clarify the specific conditions and potential mechanisms underlying the effects of physical activity interventions on physiological function and stressor-evoked responses in children and adolescents (Mücke et al., 2018).

### 4.3 Limitations

Several limitations of the present study should be acknowledged. First, the included studies varied in several characteristics of the physical activity interventions, including activity type, intensity, frequency, session duration, and intervention period, which may have resulted in differences in the actual physical activity dose received across studies and consequently increased uncertainty in the estimated effects. This issue may be particularly relevant to psychological outcomes, as different intervention types may influence perceived stress, emotional states, and anxiety through distinct pathways; thus, the overall pooled effects may not fully capture the effects of specific intervention types. Second, there was heterogeneity in the methods used to assess outcomes across the included studies. Psychological outcomes were primarily assessed using self-report scales, whereas the instruments, scoring criteria, and assessment time points varied across studies. Physiological outcomes encompassed multiple domains, including cortisol, resting heart rate, heart rate variability (HRV), and blood pressure. The broad label “blood cortisol” does not distinguish serum from plasma measurements, limiting specimen-specific interpretation and the applicability of these findings to salivary cortisol. Although the use of standardized effect sizes can reduce the influence of differences in measurement scales across instruments, it cannot completely eliminate potential bias arising from variations in measurement methods and assessment timing. In addition, different HRV indices reflect distinct aspects of autonomic regulation, and their combined interpretation therefore warrants caution. More broadly, related psychological symptoms and resting physiological measures are not specific to stress; their inclusion limits conclusions about stress reactivity or recovery. Because effect sizes were derived from unadjusted post-intervention comparisons, baseline imbalances and residual confounding, particularly in non-randomized studies, may have influenced the pooled estimates. Third, substantial statistical heterogeneity was observed for some outcomes, particularly anxiety, depressive symptoms, and several physiological indicators (Higgins et al., 2003). This suggests that the true intervention effects may not be consistent across studies. Although a random-effects model was used and the robustness of the findings was examined through sensitivity analyses and other approaches, the sources of heterogeneity could not be fully explained. Furthermore, the limited number of included studies constrained subgroup analyses or meta-regression to investigate potential moderators, such as activity type, age, sex, and intervention dose. With few studies, heterogeneity tests have limited power and I^2^ estimates are uncertain; an I^2^ of 0% does not establish the absence of clinically or methodologically meaningful heterogeneity (Higgins et al., 2003). Fourth, the evidence base for some outcomes was relatively limited. For example, fatigue was examined in only four studies, and several physiological outcomes were likewise reported in only a small number of studies; therefore, the absence of statistically significant effects does not completely rule out the possibility of true underlying effects. At the same time, some studies had relatively small sample sizes, which may have resulted in insufficient statistical power and less stable effect estimates. Accordingly, statistically non-significant findings should be interpreted as indicating that the current evidence is insufficient to establish a clear intervention effect, rather than as evidence that these interventions have no effect (Altman and Bland, 1995). Finally, although this systematic review was conducted according to prespecified search and assessment methods and the RoB 2, ROBINS-I, and GRADE frameworks were used to evaluate the quality and certainty of the evidence, the methodological quality and completeness of reporting of the included studies may still have influenced the overall findings. Dedicated Chinese-language databases were not searched, so relevant Chinese-language studies may have been missed. Language bias, publication bias, and selective reporting bias cannot be excluded, and the limited number of studies for some outcomes reduces the ability to reliably detect potential publication bias (Sterne et al., 2011). Therefore, the findings of this study should be interpreted in the context of the certainty of the evidence and the uncertainty across studies, and future randomized controlled trials with larger sample sizes, more standardized intervention protocols, more consistent outcome measurements, and longer follow-up periods are warranted to further verify the long-term effects of physical activity interventions on stress-related physiological and psychological outcomes in children and adolescents.

## 5 Conclusion

Low-certainty evidence suggested a possible small reduction in resting systolic blood pressure. Very-low-certainty evidence suggested possible reductions in perceived stress, depressive symptoms, resting diastolic blood pressure, and HRV-LF/HF; evidence for the remaining outcomes was inconclusive. Statistical significance for several outcomes depended on the inferential method or inclusion of individual studies. The non-specificity of depressive symptoms, resting blood pressure and HRV limits inferences about stress regulation. Given the substantial heterogeneity observed for some outcomes, the limited number of available studies, and certain limitations in the certainty of the evidence, these findings should be interpreted with caution. Future well-designed randomized controlled trials with clearly defined physical activity intervention protocols and larger sample sizes are warranted to further clarify the effects of different activity doses and participant characteristics on stress-related outcomes in children and adolescents.

## Data Availability

The original contributions presented in the study are included in the article/Supplementary material, further inquiries can be directed to the corresponding author.
