# Supplementary material for: The effects of physical activity interventions on stress-related psychological and physiological outcomes in children and adolescents: a systematic review and meta-analysis
Source: Front Psychol. 2026 Oct 21;17:2000159. doi: 10.3389/fpsyg.2026.2000159 (PMC13730851; doi:10.3389/fpsyg.2026.2000159)
Supplement: Supplementary file 1 [file Supplementary_file_1.docx]

**Supplementary Material**

This supplement contains Supplementary Methods S1, Tables S1-S7, and Figures S1-S7 for the systematic review and meta-analysis of physical activity interventions and stress-related psychological and physiological outcomes in children and adolescents.

# **Supplementary Methods S1. Full database search strategies**

The PubMed strategy used MeSH terms for children, adolescents, exercise, and motor activity, combined with title and abstract terms for physiological and psychological stress indicators. The Web of Science and Cochrane strategies used the corresponding free-text terms. Embase used Emtree terms and title/abstract fields.

## **PubMed**

("Child"[Mesh] OR "Adolescent"[Mesh] OR child*[Title/Abstract] OR adolescen*[Title/Abstract] OR youth*[Title/Abstract] OR teen*[Title/Abstract] OR pediatric*[Title/Abstract] OR paediatric*[Title/Abstract] OR schoolchild*[Title/Abstract]) AND ("Exercise"[Mesh] OR "Motor Activity"[Mesh] OR exercise*[Title/Abstract] OR "physical activ*"[Title/Abstract] OR "physical training"[Title/Abstract] OR "exercise training"[Title/Abstract] OR sport*[Title/Abstract] OR aerobic*[Title/Abstract] OR resistance training[Title/Abstract] OR HIIT[Title/Abstract] OR "high-intensity interval training"[Title/Abstract] OR yoga[Title/Abstract] OR qigong[Title/Abstract] OR "tai chi"[Title/Abstract]) AND (("heart rate variability"[Title/Abstract] OR HRV[Title/Abstract] OR RMSSD[Title/Abstract] OR SDNN[Title/Abstract] OR pNN50[Title/Abstract] OR cortisol[Title/Abstract] OR "salivary cortisol"[Title/Abstract] OR "cortisol awakening response"[Title/Abstract] OR "cortisol reactivity"[Title/Abstract] OR "salivary alpha-amylase"[Title/Abstract] OR "salivary amylase"[Title/Abstract] OR "perceived stress"[Title/Abstract] OR "Perceived Stress Scale"[Title/Abstract]) OR (("stress reactivity"[Title/Abstract] OR "stress response"[Title/Abstract] OR "stress recovery"[Title/Abstract] OR "psychosocial stress"[Title/Abstract] OR "psychological stress"[Title/Abstract] OR "Trier Social Stress Test"[Title/Abstract] OR TSST[Title/Abstract] OR "cold pressor"[Title/Abstract]) AND ("heart rate"[Title/Abstract] OR "blood pressure"[Title/Abstract] OR "systolic blood pressure"[Title/Abstract] OR "diastolic blood pressure"[Title/Abstract] OR cortisol[Title/Abstract] OR anxiety[Title/Abstract])))

## **Web of Science**

(child* OR adolescen* OR youth* OR teen* OR pediatric* OR paediatric* OR schoolchild*) AND (exercise* OR "physical activ*" OR "physical training" OR "exercise training" OR sport* OR aerobic* OR "resistance training" OR HIIT OR "high-intensity interval training" OR yoga OR qigong OR "tai chi") AND (("heart rate variability" OR HRV OR RMSSD OR SDNN OR pNN50 OR cortisol OR "salivary cortisol" OR "cortisol awakening response" OR "cortisol reactivity" OR "salivary alpha-amylase" OR "salivary amylase" OR "perceived stress" OR "Perceived Stress Scale") OR (("stress reactivity" OR "stress response" OR "stress recovery" OR "psychosocial stress" OR "psychological stress" OR "Trier Social Stress Test" OR TSST OR "cold pressor") AND ("heart rate" OR "blood pressure" OR "systolic blood pressure" OR "diastolic blood pressure" OR cortisol OR anxiety)))

## **Embase**

('child'/exp OR 'adolescent'/exp OR child*:ti,ab OR adolescen*:ti,ab OR youth*:ti,ab OR teen*:ti,ab OR pediatric*:ti,ab OR paediatric*:ti,ab OR schoolchild*:ti,ab) AND ('exercise'/exp OR 'physical activity'/exp OR 'exercise therapy'/exp OR exercise*:ti,ab OR 'physical activ*':ti,ab OR 'physical training':ti,ab OR 'exercise training':ti,ab OR sport*:ti,ab OR aerobic*:ti,ab OR 'resistance training':ti,ab OR hiit:ti,ab OR 'high intensity interval training':ti,ab OR yoga:ti,ab OR qigong:ti,ab OR 'tai chi':ti,ab) AND (('heart rate variability'/exp OR 'heart rate variability':ti,ab OR hrv:ti,ab OR rmssd:ti,ab OR sdnn:ti,ab OR pnn50:ti,ab OR 'cortisol'/exp OR cortisol:ti,ab OR 'salivary cortisol':ti,ab OR 'cortisol awakening response':ti,ab OR 'cortisol reactivity':ti,ab OR 'alpha amylase'/exp OR 'salivary alpha amylase':ti,ab OR 'salivary amylase':ti,ab OR 'perceived stress':ti,ab OR 'perceived stress scale':ti,ab) OR (('stress reactivity':ti,ab OR 'stress response':ti,ab OR 'stress recovery':ti,ab OR 'psychosocial stress':ti,ab OR 'psychological stress':ti,ab OR 'trier social stress test':ti,ab OR tsst:ti,ab OR 'cold pressor':ti,ab) AND ('heart rate':ti,ab OR 'blood pressure':ti,ab OR 'systolic blood pressure':ti,ab OR 'diastolic blood pressure':ti,ab OR cortisol:ti,ab OR anxiety:ti,ab)))

## **Cochrane**

(child* OR adolescen* OR youth* OR teen* OR pediatric* OR paediatric* OR schoolchild*) AND (exercise* OR "physical activ*" OR "physical training" OR "exercise training" OR sport* OR aerobic* OR "resistance training" OR HIIT OR "high-intensity interval training" OR yoga OR qigong OR "tai chi") AND (("heart rate variability" OR HRV OR RMSSD OR SDNN OR pNN50 OR cortisol OR "salivary cortisol" OR "cortisol awakening response" OR "cortisol reactivity" OR "salivary alpha-amylase" OR "salivary amylase" OR "perceived stress" OR "Perceived Stress Scale") OR (("stress reactivity" OR "stress response" OR "stress recovery" OR "psychosocial stress" OR "psychological stress" OR "Trier Social Stress Test" OR TSST OR "cold pressor") AND ("heart rate" OR "blood pressure" OR "systolic blood pressure" OR "diastolic blood pressure" OR cortisol OR anxiety)))

# **GRADE certainty of evidence**

**Table S1. GRADE certainty-of-evidence assessment for pooled outcomes.**

**A. Outcome-level judgments after the imprecision reassessment**

| **Outcome** | **k** | **N** | **Hedges’ g 95% CI** | **Risk of bias** | **Inconsistency** | **Indirectness** | **Imprecision** | **Publication bias** | **Certainty** |
| --- | --- | --- | --- | --- | --- | --- | --- | --- | --- |
| Anxiety | 15 | 1424 | -0.34 [-0.79, 0.11] | Serious | Very serious | Not serious | Serious | No clear signal | Very low ⊕◯◯◯ |
| Depressive symptoms | 13 | 1360 | -0.53 [-1.04, -0.02] | Very serious | Very serious | Not serious | Serious | No clear signal | Very low ⊕◯◯◯ |
| Perceived / general stress | 7 | 2492 | -0.28 [-0.48, -0.08] | Serious | Serious | Not serious | Serious | Not tested | Very low ⊕◯◯◯ |
| Fatigue | 4 | 383 | -0.11 [-0.32, 0.10] | Serious | Not serious | Not serious | Serious | Not tested | Low ⊕⊕◯◯ |
| Resting systolic blood pressure | 10 | 1262 | -0.15 [-0.26, -0.04] | Serious | Not serious | Not serious | Serious | No clear signal | Low ⊕⊕◯◯ |
| Resting diastolic blood pressure | 10 | 3157 | -0.33 [-0.64, -0.01] | Serious | Very serious | Not serious | Serious | Suspected | Very low ⊕◯◯◯ |
| Resting heart rate | 7 | 487 | -0.10 [-0.28, 0.08] | Serious | Not serious | Not serious | Serious | Not tested | Low ⊕⊕◯◯ |
| Blood cortisol | 4 | 90 | -0.07 [-0.80, 0.67] | Serious | Serious | Serious | Very serious | Not tested | Very low ⊕◯◯◯ |
| HRV: RMSSD | 4 | 138 | 0.03 [-0.31, 0.36] | Serious | Not serious | Serious | Very serious | Not tested | Very low ⊕◯◯◯ |
| HRV: SDNN | 4 | 138 | 0.01 [-0.32, 0.35] | Serious | Not serious | Serious | Very serious | Not tested | Very low ⊕◯◯◯ |
| HRV: LF/HF | 4 | 167 | -0.43 [-0.80, -0.05] | Serious | Not serious | Serious | Serious | Not tested | Very low ⊕◯◯◯ |

Serious = one level downgraded; very serious = two levels downgraded; not serious = no downgrade. Ratings start at high certainty, with limitations of randomized and ROBINS-I-assessed non-randomized evidence considered under risk of bias. Final certainty cannot fall below very low. No upgrading was applied. N is the unadjusted number analyzed for each outcome, not the number of unique participants across the review. The existing judgments for domains other than imprecision were retained; their reasons are detailed in panel D.

**Table S1 (continued). B. Operational criteria and scope of the reassessment**

Target and rationale. The post hoc reassessment concerned uncertainty in standardized effect magnitude, assessed separately for each outcome. The reference |g| = 0.20 follows the conventional small-SMD interpretation described in the Cochrane Handbook, which also cautions that clinical importance depends on context (3). It was chosen to make judgments on the common standardized scale transparent and consistent, not to equate clinical importance across outcomes. Outcome-specific MIDs were neither prespecified nor established for the measures and populations in this review; this does not imply that relevant MIDs are unavailable. The benchmark is therefore a distribution-based fallback, not a GRADE-recommended universal MID. The judgments remain conditional on this choice, and another defensible threshold could change them. The primary estimates, study sets and sensitivity analyses were unchanged.

Imprecision. Following the GRADE emphasis on confidence intervals relative to explicit thresholds (2), each primary 95% CI was compared with −0.20 and +0.20. The operational rule for this review was a one-level downgrade when the CI included values within this central range and beyond one boundary, and a two-level downgrade when it extended beyond both boundaries. These are statistical magnitude categories, not validated benefit/harm thresholds. Study count, I² = 0%, crossing zero, or a change in P-value category was insufficient alone. OIS was supporting information and did not add another downgrade for uncertainty already reflected in the CI.

Information size. The OIS used a normal approximation for two independent groups with equal allocation and common variance (4, 5). With two-sided α = 0.05, power 1 − β = 0.80, and standardized difference δ = Δ/σ = 0.20, n per group = 2 × (1.960 + 0.8416)² / 0.20² = 392.44, rounded up to 393; total OIS = 786. Here Δ is the assumed between-group mean difference and σ the common population SD; 1.960 and 0.8416 are the corresponding normal quantiles. All 11 outcomes used these same assumptions, not their observed pooled effects. This is a nominal two-group planning reference, not an effective meta-analysis sample size. No allowance was made for unequal allocation, clustering or heterogeneity, which may increase information requirements; nominal N ≥ 786 therefore does not by itself establish adequate information.

Risk of bias. The previously agreed outcome-level judgments were retained, considering the contribution of RoB 2 and ROBINS-I assessments. One-level deductions reflect important concerns about allocation, outcome measurement, missing data, reporting or analysis; the existing two-level judgment for depressive symptoms is explained in panel D. Non-randomization was not counted again as a separate starting-level penalty. Clustering and transformation concerns were handled as limitations of the primary analyses, without inventing corrected effect estimates.

Inconsistency. Effect direction and magnitude, overlap of study confidence intervals, I² and τ² were considered together. Very serious ratings apply to anxiety, depressive symptoms and diastolic blood pressure, where I² exceeded 90% and effects differed substantially. Perceived/general stress and blood cortisol retained one-level deductions. A zero I² estimate or a change in leave-one-out statistical significance was not treated as proof of consistency or inconsistency.

Indirectness and reporting bias. Existing indirectness judgments for cortisol and HRV were retained for the stress-related interpretation of these biomarkers; they are explained separately in panel D. This does not turn resting biomarkers into direct measures of psychological stress. Publication bias was downgraded only for diastolic blood pressure, where Egger asymmetry was present; this remains suggestive, not proof of publication bias. For k < 10, lack of an Egger test did not itself trigger downgrading. Non-significant Egger tests do not establish absence of bias.

**Table S1 (continued). C. Explicit imprecision judgments**

| **Outcome** | **N** | **95% CI** | **CI width** | **N / 786** | **Threshold interpretation** | **Imprecision** |
| --- | --- | --- | --- | --- | --- | --- |
| Anxiety | 1424 | -0.788 to 0.108 | 0.896 | 1.81 | Spans the central range and reductions beyond −0.20. | Serious |
| Depressive symptoms | 1360 | -1.042 to -0.023 | 1.019 | 1.73 | Spans the central range and reductions beyond −0.20. | Serious |
| Perceived / general stress | 2492 | -0.479 to -0.078 | 0.401 | 3.17 | Spans the central range and reductions beyond −0.20. | Serious |
| Fatigue | 383 | -0.316 to 0.098 | 0.414 | 0.49 | Spans the central range and reductions beyond −0.20. | Serious |
| Resting systolic blood pressure | 1262 | -0.261 to -0.038 | 0.223 | 1.61 | Spans the central range and reductions beyond −0.20. | Serious |
| Resting diastolic blood pressure | 3157 | -0.645 to -0.014 | 0.631 | 4.02 | Spans the central range and reductions beyond −0.20. | Serious |
| Resting heart rate | 487 | -0.283 to 0.079 | 0.362 | 0.62 | Spans the central range and reductions beyond −0.20. | Serious |
| Blood cortisol | 90 | -0.803 to 0.672 | 1.474 | 0.11 | Spans effects below −0.20 and above +0.20. | Very serious |
| HRV: RMSSD | 138 | -0.308 to 0.364 | 0.671 | 0.18 | Spans effects below −0.20 and above +0.20. | Very serious |
| HRV: SDNN | 138 | -0.321 to 0.351 | 0.672 | 0.18 | Spans effects below −0.20 and above +0.20. | Very serious |
| HRV: LF/HF | 167 | -0.805 to -0.046 | 0.759 | 0.21 | Spans the central range and reductions beyond −0.20. | Serious |

The CI width is reported descriptively; there is no universal GRADE cutoff for a “narrow” interval. Direction is intervention minus control. In the HRV rows, opposite numerical directions are not automatically labeled benefit and harm. OIS comparisons are nominal and supportive; no extra level was deducted merely because N < 786.

Focused checks. For fatigue, the interval includes reductions greater than 0.20 SD as well as no difference or a small increase, despite its modest numerical width; the one-level deduction is not based on k = 4 or I² = 0%. Resting heart rate has the same interpretive limitation. For systolic blood pressure, both CI limits are negative, but the interval crosses the magnitude benchmark; the imprecision deduction concerns uncertainty about magnitude, not absence of statistical significance.

Systolic blood pressure leave-one-out results: omitting Domaradzki 2025 gave g = −0.145 (95% CI −0.318 to 0.029); omitting Larsen 2020 gave −0.117 (−0.240 to 0.007); and omitting Sharma 2017 gave −0.156 (−0.327 to 0.014). Across all ten omissions, g ranged from −0.182 to −0.117. These findings support cautious interpretation but do not establish directional inconsistency or justify a second penalty for the same uncertainty.

**Table S1 (continued). D. Reasons for the remaining domain judgments**

| **Outcome** | **Risk of bias** | **Inconsistency** | **Indirectness** | **Publication bias** |
| --- | --- | --- | --- | --- |
| Anxiety | Serious: six randomized entries were high risk and Norris was serious under ROBINS-I; concerns included psychological outcome measurement, attrition and confounding. | Very serious: I² = 93.7%; marked differences in size and direction. | Not serious for the named anxiety outcome. | No additional deduction; Egger test did not show clear asymmetry. |
| Depressive symptoms | Very serious, retained from the original assessment: five randomized entries were high risk, including Arslan and Sharma with large negative estimates; unblinded symptom measurement and analysis/reporting concerns materially limit confidence. | Very serious: I² = 94.8%; marked dispersion of estimates. | Not serious for the named symptom outcome. | No additional deduction; no clear Egger signal. |
| Perceived / general stress | Serious: concerns in the randomized evidence and confounding in Norris; analysis of a large cluster trial also limits confidence. | Serious: I² = 49.9%; effects differ and the Ranjani estimate materially influences the synthesis. | Not serious for the named stress outcome. | Not tested (k < 10); no automatic deduction. |
| Fatigue | Serious: Khalsa was high risk; the remaining three entries had some concerns, including participant-reported outcomes and analysis/reporting limitations. | Not serious: I² = 0%; no clear statistical inconsistency, with limited ability to detect it. | Not serious for the named fatigue outcome. | Not tested (k < 10); no automatic deduction. |
| Resting systolic blood pressure | Serious: randomized entries had some concerns; Norris was serious under ROBINS-I; unadjusted cluster analyses were an additional limitation. | Not serious: primary I² = 0%; leave-one-out estimates stayed negative. | Not serious for resting blood pressure itself; this does not establish stress-regulation benefit. | No additional deduction; no clear Egger signal. |
| Resting diastolic blood pressure | Serious: concerns in randomized entries, confounding in Norris, and cluster-analysis limitations. | Very serious: I² = 90.5%; large differences between study estimates. | Not serious for resting blood pressure itself. | One-level deduction: Egger P = 0.034; asymmetry may also arise from heterogeneity or SMD–SE coupling. |
| Resting heart rate | Serious: randomized entries had some concerns; Norris had serious risk of confounding/selection bias; cluster-analysis limitations remain. | Not serious: I² = 0%; no clear statistical inconsistency. | Not serious for resting heart rate itself. | Not tested (k < 10); no automatic deduction. |
| Blood cortisol | Serious: three randomized entries had some concerns and Gorostiaga had serious ROBINS-I risk. | Serious: I² = 66.3%; estimates differ in direction. | Serious, retained: serum/plasma collection and training contexts constrain a common stress-regulation interpretation of the measurements. | Not tested (k < 10); no automatic deduction. |
| HRV: RMSSD | Serious: three randomized entries had some concerns; Ivaki had serious ROBINS-I risk. Analysis/scale-transformation assumptions limit confidence. | Not serious: I² = 0%; only four studies, so heterogeneity remains uncertain. | Serious, retained: resting HRV and differences in recording/timing are indirect for the broader stress-regulation interpretation. Transformation assumptions belong to risk of bias, not an extra indirectness penalty. | Not tested (k < 10); no automatic deduction. |
| HRV: SDNN | Serious: three randomized entries had some concerns; Ivaki had serious ROBINS-I risk. Analysis/scale-transformation assumptions limit confidence. | Not serious: I² = 0%; only four studies, so heterogeneity remains uncertain. | Serious, retained: resting HRV and differences in recording/timing are indirect for the broader stress-regulation interpretation; no duplicate penalty for transformation assumptions. | Not tested (k < 10); no automatic deduction. |
| HRV: LF/HF | Serious: three randomized entries had some concerns; Ivaki had serious ROBINS-I risk. | Not serious: I² = 29.2%. Fragile leave-one-out significance is not a separate inconsistency deduction. | Serious, retained: LF/HF has limited specificity as an indicator of stress regulation; a lower ratio does not by itself establish improved autonomic balance. | Not tested (k < 10); no automatic deduction. |

Interpretive scope. The retained indirectness judgments concern inference about stress regulation from cortisol/HRV, whereas the numerical effects remain outcome-specific. These judgments should not be read as evidence that all included outcomes measure the same construct. The present revision did not repeat the study-level risk-of-bias assessment or change the agreed RoB 2/ROBINS-I ratings.

**Methodological references**

1. Schünemann HJ, Higgins JPT, Vist GE, et al. Chapter 14: Completing “Summary of findings” tables and grading the certainty of the evidence. Cochrane Handbook for Systematic Reviews of Interventions. https://www.cochrane.org/authors/handbooks-and-manuals/handbook/current/chapter-14

2. Zeng L, Brignardello-Petersen R, Hultcrantz M, et al. GRADE Guidance 34: update on rating imprecision using a minimally contextualized approach. J Clin Epidemiol. 2022;150:216–224. doi:10.1016/j.jclinepi.2022.07.014.

3. Schünemann HJ, Vist GE, Higgins JPT, et al. Chapter 15: Interpreting results and drawing conclusions. Cochrane Handbook for Systematic Reviews of Interventions. Version 6.5. Cochrane; 2024. Section 15.5.3.1. https://www.cochrane.org/authors/handbooks-and-manuals/handbook/current/chapter-15

4. Guyatt GH, Oxman AD, Kunz R, Brozek J, Alonso-Coello P, Rind D, et al. GRADE guidelines 6. Rating the quality of evidence—imprecision. J Clin Epidemiol. 2011;64(12):1283–1293. doi:10.1016/j.jclinepi.2011.01.012.

5. Guyatt G, Oxman AD, Kunz R, Brozek J, Alonso-Coello P, Rind D, et al. Corrigendum to GRADE guidelines 6. Rating the quality of evidence-imprecision. J Clin Epidemiol 2011;64:1283–1293. J Clin Epidemiol. 2021;137:265. doi:10.1016/j.jclinepi.2021.04.014.

**Table S2. Database-specific search yields before deduplication.**

| **Database** | **Search records** |
| --- | --- |
| PubMed | 2,204 |
| Web of Science | 1,951 |
| Embase | 1,500 |
| Cochrane Library | 991 |
| Total before deduplication | 6,646 |

*Note. Counts are the records returned by the final searches before deduplication and screening.*

# **Study characteristics**

**Table S3. Characteristics of the 35 reports included in the quantitative synthesis.**

| **Meta-analysis ID** | **Country** | **Participants and background** | **Design** | **Duration** | **Physical activity intervention** | **Comparator** | **Contributed outcomes** |
| --- | --- | --- | --- | --- | --- | --- | --- |
| Akgul2020 | Germany | Preadolescent children aged 9–10 years (71 randomized) | RCT | 10 weeks; 3 sessions/week | Cardiovascular exercise training or motor exercise training | Assisted homework | ANX |
| Arslan2026 | Türkiye | High-school students; mean age 16.9 ± 0.9 years | Cluster RCT | 4 weeks | Yoga-based cognitive-behavioral stress-management program | Brief stress education | ANX; DEP |
| Bazzano2022 | United States | Students in grades 7–8, including students with IEP/504 plans | Cluster RCT | 8 weeks | Yoga and mindfulness | Attention control | ANX; DEP |
| Butzer2016 | United States | Grade 7 students | Cluster RCT | 32 classes | School-based yoga | Physical education as usual | DEP; PSS; FATIGUE |
| Chang2013 | Taiwan, China | Grade 7 students from two classes (N=67) | Cluster RCT | 8 weeks | Laughing Qigong Program | Reading/homework activities | SBP; DBP; HR; LF/HF |
| Crews2004 | United States | Low-income Hispanic fourth-grade children; 33 boys and 33 girls | RCT | 6 weeks | Aerobic activities including cycling, running, and trampoline exercise | Low-intensity physical activity | ANX; DEP |
| Darragi2026 | Tunisia | Elite female soccer players; mean age 15.3 ± 0.9 years | RCT | 12 weeks; 2 sessions/week | Additional resistance training plus usual soccer training | Usual soccer training | Blood cortisol |
| Domaradzki2025 | Poland | School adolescents | Cluster RCT | 12 weeks plus 8-week follow-up | School-based HIIT or high-intensity power training | Usual physical education | SBP; DBP |
| Gamelin2009 | France | Tanner stage I, non-obese children; mean age 9.6 ± 1.2 years | RCT | 7 weeks; 3 × 30 min/week | Maximal and supramaximal interval running | Usual activities | HR; RMSSD; SDNN |
| Gorostiaga1999 | Spain | Male adolescent handball players aged 14–16 years | Non-randomized intervention study | 6 weeks; 2 sessions/week | Additional heavy resistance training plus handball training | Usual handball training | Blood cortisol |
| Hagins2013 | United States | Grade 6 students aged 10–11 years | RCT | 15 weeks | Yoga | Physical education | SBP; DBP; HR |
| Hammami2017 | Tunisia | Untrained healthy boys; mean age 15.9 ± 0.6 years | RCT | 8 weeks; 2 × 30–45 min/week | Small-sided recreational soccer | Usual activities | HR; RMSSD; SDNN; LF/HF |
| Ivaki2021 | Germany | Primary-school children aged 8–12 years; ECG subsample | Non-randomized intervention study | 16 weeks; 45 min/week plus brief daily practice | YoBEKA yoga and mindfulness | Usual curriculum | RMSSD; SDNN; LF/HF |
| Jeon2015 | South Korea | Healthy middle-school students; approximately 15 years old | RCT | 8 weeks; 3 sessions/week | Treadmill aerobic exercise (200 kcal/session) | Usual activities | Blood cortisol |
| Ketelhut2020 | Germany | Children; mean age 8.2 ± 0.6 years | Cluster RCT | 37 weeks; 2 additional 45-min sessions/week | Additional school-based exercise | Usual physical education (3 × 45 min/week) | SBP |
| Ketelhut2024 | Germany | Typically developing students; mean age 11.0 ± 0.7 years | Cluster RCT | 12 weeks; 2 × 20 min/week | HIIT incorporated into physical education | Usual physical education | SBP; DBP; HR; RMSSD; SDNN; LF/HF |
| Khalsa2011 | United States | Secondary-school students aged approximately 16–17 years | Cluster RCT | 11 weeks; 30–40 min/session | Yoga Ed | Physical education as usual | FATIGUE |
| Khalsa2012 | United States | Secondary-school students aged 15–19 years | Cluster RCT | 11 weeks; 2–3 sessions/week | Yoga Ed | Physical education as usual | ANX; DEP |
| Larsen2020 | Denmark | Healthy children aged 8–10 years | Cluster RCT | 10 months; 12 min/day | Small-sided games and interval running; two intervention arms combined | Usual activities | SBP; DBP; HR |
| Meng2022 | China | Boys with obesity aged 10–13 years; mean age 11.2 ± 0.7 years | RCT | 12 weeks | HIIT and moderate-intensity continuous training; arms combined | Non-exercise control | SBP; DBP |
| Mona2024 | India | Adolescents with excessive screen-use exposure | RCT | 12 weeks; 3 × 45 min/week | Integrated yoga | Digital health education | ANX; DEP |
| Murray2022 | United States | Adolescents (N=42) | RCT | 3 weeks; 5 × 10 min/week | BOXVR and guided YouTube boxing; arms combined | No-intervention control | ANX; PSS |
| Noggle2012 | United States | Students in grades 11–12 | Cluster RCT | 10 weeks; 2–3 sessions/week | Kripalu-based yoga | Physical education as usual | DEP; PSS; FATIGUE |
| Norris1992 | United Kingdom | Adolescents aged approximately 16–17 years | Non-randomized intervention study | 10 weeks | High- and moderate-intensity aerobic exercise plus flexibility; intervention arms combined | Non-exercise control at a neighboring school | ANX; DEP; PSS; SBP; DBP; HR |
| Ouerghi2026 | Tunisia | Healthy, untrained boys aged 14–16 years | RCT | 10 weeks; 3 sessions/week | High-intensity interval running | Non-training control | ANX; DEP; PSS; Blood cortisol |
| Parajuli2022 | India | Female schoolchildren aged 12–14 years with low academic performance | RCT | 2 months | Yoga | Physical exercise | ANX |
| Philippot2019 | Belgium | Non-clinical children aged 9–11 years | RCT | 5 weeks | High-intensity exercise | Low- to moderate-intensity exercise | ANX; DEP |
| Quach2016 | United States | Students aged 12–17 years | RCT | 4 weeks | Hatha yoga arm | Wait-list control | ANX; PSS |
| Ranjani2023 | India | Students aged 13–15 years from public and private schools | Cluster RCT | Approximately 5 months; 17 × 45-min sessions | School-based yoga | Health education | PSS; DBP |
| Richards2014 | Uganda | Boys aged 11–14 years in the randomized component | RCT | 11-week soccer league | Sport-for-development soccer intervention | Wait-list control | ANX; DEP |
| Rodrigues2021 | Portugal | Students aged 13–18 years in grades 7–10 | RCT | 6 weeks | Qigong | Documentary viewing/usual study activities; control arms combined | ANX |
| Sarabia2015 | Spain | Male tennis players; mean age 15 ± 1 years; approximately 5 years of training | RCT | 6-week main program plus 1-week taper | Non-failure resistance training plus usual tennis training | Usual tennis training | FATIGUE |
| Sharma2013 | India | Grade 10 boys aged 16–17 years with high examination anxiety | RCT | 6 weeks | Yoga postures and breathing exercises | Usual curriculum | DEP |
| Sharma2017 | India | Healthy adolescents aged 12–17 years, including athlete and non-athlete strata | RCT | 6 months | Structured physical-activity training | Unstructured physical activity | SBP; DBP |
| Silva2025 | Brazil | Students aged 12–15 years; boys and girls (N=306) | Cluster RCT | 12 weeks | ActTeens school-based physical-activity intervention | Usual curriculum | ANX; DEP |
| Yu2016 | Hong Kong, China | Non-obese physically active boys and girls aged 11–13 years | RCT | 10 weeks; 2 sessions/week | Resistance training | Usual physical education/activity | SBP; DBP |

*Note. Thirty-five unique reports were included. The Khalsa report is displayed as two outcome-specific analysis IDs because it contributed distinct outcome sets; it contributed no more than one effect estimate to any single outcome. Abbreviations: ANX, anxiety; COR, blood cortisol; DBP, resting diastolic blood pressure; DEP, depressive symptoms; FAT, fatigue; HR, resting heart rate; HRV, heart rate variability; PSS, perceived or general stress; SBP, resting systolic blood pressure.*

# **Risk of bias assessment**

Randomized studies were assessed with RoB 2 and non-randomized intervention studies with ROBINS-I. The traffic-light figures show outcome-level judgments; the combined domain strip summarizes one rating per meta-analysis study ID within each applicable domain. All 36 retained analysis IDs were assessed from the corresponding local full-text reports. These IDs represent 35 unique publications because Khalsa2011 and Khalsa2012 map to the same report. Grey circles in the RoB 2 figure indicate that the cluster-recruitment domain is not applicable to individually randomized trials; they do not indicate missing information.

**Figure S1.** Overall risk-of-bias domain summary for randomized and non-randomized studies assessed using RoB 2 and ROBINS-I.

**Figure S2.** Study-level RoB 2 judgments for randomized studies.

**Figure S3.** Study-level ROBINS-I judgments for non-randomized intervention studies.

# **Prediction intervals**

**Table S4. Pooled effects and 95% prediction intervals for the 11 outcomes.**

Prediction intervals were added using the same studies, Hedges’ g estimates, sampling variances and REML models as the primary analyses. The pooled estimates and their original normal-theory confidence intervals are reproduced for comparison.

| **Outcome** | **k** | **Hedges’ g** | **95% CI** | **I² (%)** | **τ²** | **95% PI** |
| --- | --- | --- | --- | --- | --- | --- |
| Anxiety | 15 | −0.340 | [−0.788, 0.108] | 93.7 | 0.717 | [−2.234, 1.554] |
| Depressive symptoms | 13 | −0.532 | [−1.042, −0.023] | 94.8 | 0.809 | [−2.593, 1.528] |
| Perceived / general stress | 7 | −0.279 | [−0.479, −0.078] | 49.9 | 0.030 | [−0.794, 0.236] |
| Fatigue | 4 | −0.109 | [−0.316, 0.098] | 0.0 | 0.000 | [−0.563, 0.345] |
| Resting systolic blood pressure | 10 | −0.150 | [−0.261, −0.038] | 0.0 | 0.000 | [−0.281, −0.019] |
| Resting diastolic blood pressure | 10 | −0.329 | [−0.645, −0.014] | 90.5 | 0.201 | [−1.429, 0.771] |
| Resting heart rate | 7 | −0.102 | [−0.283, 0.079] | 0.0 | 0.000 | [−0.339, 0.136] |
| Blood cortisol | 4 | −0.065 | [−0.803, 0.672] | 66.3 | 0.373 | [−3.153, 3.022] |
| HRV: RMSSD | 4 | 0.028 | [−0.308, 0.364] | 0.0 | 0.000 | [−0.709, 0.765] |
| HRV: SDNN | 4 | 0.015 | [−0.321, 0.351] | 0.0 | 0.000 | [−0.723, 0.752] |
| HRV: LF/HF | 4 | −0.425 | [−0.805, −0.046] | 29.2 | 0.044 | [−1.653, 0.803] |

Note. CI, confidence interval; PI, prediction interval; k, number of contributing studies; τ², REML estimate of between-study variance. Negative g values indicate lower measured values in the intervention group; they do not uniformly indicate benefit across outcomes.

Method. Approximate 95% PIs were calculated using the pooled mean, its conventional standard error, the REML estimate of τ², and a t-distribution with k−2 degrees of freedom (Riley et al., 2011). PIs describe the range of underlying effects in a new study sufficiently similar to those included, assuming normally distributed study effects. They are not intervals for an individual participant or for the observed estimate in a future study. The 95% CIs retain the original normal-theory method; the t-based PI calculation does not replace primary inference.

Caution. All 11 PIs are reported for completeness. The five outcomes with k=4 (fatigue, blood cortisol, RMSSD, SDNN and LF/HF) are exploratory because both heterogeneity and PIs are poorly estimated with so few studies. A τ² estimate close to zero does not establish homogeneity. The diastolic blood pressure PI requires additional caution because of funnel-plot asymmetry (Table S7). PIs remain approximate and do not correct bias or explain heterogeneity.

Interpretation. Only the systolic blood pressure PI excludes zero (−0.281 to −0.019); this conditional result does not establish a uniform effect across settings. All other PIs include zero. The wide intervals for anxiety, depressive symptoms and diastolic blood pressure limit extrapolation of their pooled average effects to a new setting.

Reference: Riley RD, Higgins JPT, Deeks JJ. Interpretation of random effects meta-analyses. BMJ. (2011) 342:d549. doi:10.1136/bmj.d549.

# **Additional analysis of statistical inference**

**Table S5. Comparison of normal-theory and modified Hartung–Knapp inference for REML random-effects meta-analyses.**

All 11 outcomes were reanalysed using the same studies, Hedges’ g estimates, sampling variances and REML heterogeneity estimator as the primary analysis. Only the method used to calculate the pooled-effect confidence interval and P value was changed.

| **Outcome** | **k** | **Hedges’ g** | **Normal-theory 95% CI** | **P** | **Modified HK 95% CI** | **P** |
| --- | --- | --- | --- | --- | --- | --- |
| Anxiety | 15 | −0.340 | [−0.7880, 0.1083] | 0.1371 | [−0.8344, 0.1546] | 0.1626 |
| Depressive symptoms | 13 | −0.532 | [−1.0417, −0.0229] | 0.0406 | [−1.1015, 0.0369] | 0.0643 |
| Perceived / general stress | 7 | −0.279 | [−0.4790, −0.0782] | 0.0064 | [−0.5287, −0.0285] | 0.0344 |
| Fatigue | 4 | −0.109 | [−0.3156, 0.0982] | 0.3030 | [−0.4446, 0.2272] | 0.3788 |
| Resting systolic blood pressure | 10 | −0.150 | [−0.2613, −0.0382] | 0.0085 | [−0.2992, −0.0003] | 0.0496 |
| Resting diastolic blood pressure | 10 | −0.329 | [−0.6445, −0.0137] | 0.0409 | [−0.6932, 0.0350] | 0.0712 |
| Resting heart rate | 7 | −0.102 | [−0.2827, 0.0793] | 0.2708 | [−0.3562, 0.1528] | 0.3659 |
| Blood cortisol | 4 | −0.065 | [−0.8026, 0.6718] | 0.8619 | [−1.2975, 1.1666] | 0.8765 |
| HRV: RMSSD | 4 | 0.028 | [−0.3079, 0.3635] | 0.8710 | [−0.5173, 0.5729] | 0.8814 |
| HRV: SDNN | 4 | 0.015 | [−0.3211, 0.3509] | 0.9308 | [−0.5307, 0.5604] | 0.9363 |
| HRV: LF/HF | 4 | −0.425 | [−0.8047, −0.0459] | 0.0280 | [−1.1974, 0.3468] | 0.1779 |

Note. HK, Hartung–Knapp; CI, confidence interval; k, number of contributing studies. Both methods yield identical pooled g and τ². Modified HK uses a t distribution with k−1 degrees of freedom and an ad hoc variance adjustment constrained not to reduce the conventional standard error (Röver et al., 2015). Reanalysis: R 4.6.1, metafor 5.0-1, method="REML", test="adhoc". Four decimal places are retained to show the systolic blood pressure CI upper limit accurately.

Interpretation. Depressive symptoms, diastolic blood pressure and LF/HF have CIs that include zero under modified HK. Perceived/general stress and systolic blood pressure retain CIs below zero, although the latter is close to the threshold (P=0.0496). For anxiety, depressive symptoms and diastolic blood pressure, substantial heterogeneity limits the generalizability of the pooled average. Changing the inferential method does not explain that heterogeneity; the limited study counts also constrain reliable moderator analyses.

Reference: Röver C, Knapp G, Friede T. Hartung-Knapp-Sidik-Jonkman approach and its modification for random-effects meta-analysis with few studies. BMC Med Res Methodol. (2015) 15:99. doi:10.1186/s12874-015-0091-1.

# **Sensitivity analysis**

**Table S6. Leave-one-out sensitivity analyses of pooled outcome estimates.**

| **Outcome** | **k** | **Pooled Hedges g 95% CI** | **P** | **LOO P range** | **Significance-changing omission** |
| --- | --- | --- | --- | --- | --- |
| Anxiety | 15 | -0.340 [-0.788, 0.108] | 0.1371 | 0.1052-0.1880 | None |
| Depressive symptoms | 13 | -0.532 [-1.042, -0.023] | 0.0406 | 0.0204-0.0974 | Arslan2026; Bazzano2022; Crews2004; Khalsa2012; Noggle2012; Sharma2013 |
| Perceived / general stress | 7 | -0.279 [-0.479, -0.078] | 0.0064 | 0.0015-0.0717 | Ranjani2023 |
| Fatigue | 4 | -0.109 [-0.316, 0.098] | 0.3030 | 0.1869-0.5380 | None |
| Resting systolic blood pressure | 10 | -0.150 [-0.261, -0.038] | 0.0085 | 0.0019-0.1027 | Domaradzki2025; Larsen2020; Sharma2017 |
| Resting diastolic blood pressure | 10 | -0.329 [-0.645, -0.014] | 0.0409 | 0.0097-0.0973 | Hagins2013; Larsen2020; Meng2022; Norris1992; Ranjani2023; Sharma2017 |
| Resting heart rate | 7 | -0.102 [-0.283, 0.079] | 0.2708 | 0.1345-0.5807 | None |
| Blood cortisol | 4 | -0.065 [-0.803, 0.672] | 0.8619 | 0.3789-0.9553 | None |
| HRV: RMSSD | 4 | 0.028 [-0.308, 0.364] | 0.8710 | 0.5437-0.9276 | None |
| HRV: SDNN | 4 | 0.015 [-0.321, 0.351] | 0.9308 | 0.6311-0.8025 | None |
| HRV: LF/HF | 4 | -0.425 [-0.805, -0.046] | 0.0280 | 0.0772-0.1357 | Chang2013; Hammami2017; Ivaki2021; Ketelhut2024 |

*Note. Primary models used REML random effects with normal (Z) inference. Leave-one-out (LOO) analyses refitted the model after omitting each study in turn. Significance-changing omissions indicate statistical fragility and are not, by themselves, grounds for excluding a study.*

# **Small study effects and funnel plots**

Egger regression was prespecified for outcomes with k >= 10. The test was treated as exploratory because standardized mean differences can show effect-size-standard-error coupling and substantial heterogeneity can generate funnel-plot asymmetry.

**Table S7. Egger regression tests for small-study effects.**

| **Outcome** | **k** | **Egger statistic** | **P** |
| --- | --- | --- | --- |
| Anxiety | 15 | -0.997 | 0.337 |
| Depressive symptoms | 13 | -1.337 | 0.208 |
| Resting systolic blood pressure | 10 | 0.222 | 0.830 |
| Resting diastolic blood pressure | 10 | 2.544 | 0.034 |

**Figure S4.** Funnel plot for anxiety.

**Figure S5.** Funnel plot for depressive symptoms.

**Figure S6.** Funnel plot for resting systolic blood pressure.

**Figure S7.** Funnel plot for resting diastolic blood pressure.

*Note. The p = 0.034 result for resting diastolic blood pressure indicates funnel-plot asymmetry in this dataset but does not establish publication bias. It should be interpreted together with design differences, heterogeneity, selective reporting, and the small number of studies.*
